# Association of Depression and Anxiety With the Accumulation of Chronic Conditions

**DOI:** 10.1001/jamanetworkopen.2022.9817

**Published:** 2022-05-02

**Authors:** William V. Bobo, Brandon R. Grossardt, Sanya Virani, Jennifer L. St Sauver, Cynthia M. Boyd, Walter A. Rocca

**Affiliations:** 1 Department of Psychiatry and Psychology, Mayo Clinic Florida, Jacksonville; 2 Division of Clinical Trials and Biostatistics, Department of Quantitative Health Sciences, Mayo Clinic, Rochester, Minnesota; 3 Department of Psychiatry and Human Behavior, Warren Alpert School of Medicine, Brown University, Providence, Rhode Island; 4 Division of Epidemiology, Department of Quantitative Health Sciences, Mayo Clinic, Rochester, Minnesota; 5 The Robert D. and Patricia E. Kern Center for the Science of Health Care Delivery, Mayo Clinic, Rochester, Minnesota; 6 Division of Geriatric Medicine and Gerontology, Department of Medicine, Johns Hopkins University, Baltimore, Maryland; 7 Department of Neurology, Mayo Clinic, Rochester, Minnesota; 8 Women’s Health Research Center, Mayo Clinic, Rochester, Minnesota

## Abstract

**Importance:**

Longitudinal associations between comorbid depression and anxiety with the accumulation of chronic illnesses are unclear, and questions remain about the contributions associated with each condition in the increasing prevalence of multimorbidity.

**Objective:**

To compare the risk and rate of accumulating chronic conditions in people with depression, anxiety, and comorbid depression and anxiety vs individuals with neither depression nor anxiety.

**Design, Setting, and Participants:**

This cohort study used the Rochester Epidemiology Project medical records-linkage system to identify residents of Olmsted County, Minnesota, from January 1, 2005, to December 31, 2014, with follow-up ending December 31, 2017. The sample was divided into cohorts anchored at birthday ages of 20, 40, and 60 years. Individuals were classified at anchoring birthday age as having depression alone, anxiety alone, comorbid depression and anxiety, or neither depression nor anxiety (reference group), using electronically extracted diagnosis codes from the *International Classification of Diseases, Ninth Revision* (*ICD-9*) in the 5 years before each anchoring birthday. Data were analyzed from August 2020 through November 2021.

**Exposures:**

Depression alone, anxiety alone, comorbid depression and anxiety, or neither depression nor anxiety (reference group).

**Main Outcomes and Measures:**

The main outcome was sex-specific risk, calculated as hazard ratios (HRs) and rates of accumulation, calculated as mean annual incidence rates per 100 person-years, of 15 common chronic conditions within each birthday age cohort through the end of study.

**Results:**

Among the 40 360 individuals included across all 3 age cohorts, 21 516 (53.3%) were women. After balancing cohorts on race, Hispanic ethnicity, education level, body mass index, smoking status, and calendar year at index birthday, the risk of accumulating chronic conditions was significantly increased among women with depression alone (cohort aged 20 years: HR, 1.20 [95% CI, 1.02-1.42]; cohort aged 40 years: HR, 1.20 [95% CI, 1.10-1.31]; cohort aged 60 years: HR, 1.09 [95% CI, 1.02-1.16]) and women with comorbid depression and anxiety (cohort aged 20 years: HR, 1.60 [95% CI, 1.28-1.99]; cohort aged 40 years: HR, 1.41 [95% CI, 1.21-1.65]; cohort aged 60 years: HR, 1.29 [95% CI, 1.15-1.44]) compared with referent women in the same birthday cohorts and in men with comorbid depression and anxiety compared with referent men in the cohort aged 20 years (HR, 1.77 [95% CI, 1.08-2.91]). For women, the rates of accumulation of conditions were significantly higher across birthday cohorts in the comorbid depression and anxiety group compared with the depression alone group (eg, cohort aged 20 years: difference, 1.2 [95% CI, 0.2-2.1] per 100 person-years) and reference group (eg, cohort aged 20 years: difference, 1.7 [95% CI, 0.9-2.6] per 100 person-years). For men, compared with the reference group, the rates of accumulation of conditions were significantly higher in men with comorbid depression and anxiety in the cohort aged 20 years (difference, 1.4 [95% CI, 0.1-2.6] per 100 person-years) and in men with depression in the cohort aged 40 years (difference, 2.0 [95% CI, 0.8-3.2] per 100 person-years).

**Conclusions and Relevance:**

In this cohort study, the risk of accumulating chronic conditions was increased with depression and comorbid depression and anxiety in women across the age span and in younger men with comorbid depression and anxiety. Compared with women without depression or anxiety, there was a more rapid rate of accumulation of chronic conditions in women with depression and anxiety individually and an even higher rate when depression and anxiety cooccurred.

## Key Points

**Question:**

Are depression and/or anxiety associated with higher risk and higher rates of accumulating chronic conditions than having neither condition?

**Findings:**

In this cohort study including 40 360 individuals, the risk of accumulating chronic conditions was significantly higher in women with depression and comorbid depression and anxiety in each of 3 age cohorts (anchored at the 20th, 40th, and 60th birthdays) compared with individuals without depression or anxiety, with similar observations for men in the cohort aged 20 years. Rates of accumulation of chronic conditions were highest for women and men with combined depression and anxiety.

**Meaning:**

These findings suggest that depression and anxiety may be associated with higher rates of acquiring chronic conditions and that these associations may be magnified when depression and anxiety cooccur.

## Teaser

This cohort study assesses the risk and rate of accumulating chronic conditions in people with depression, anxiety, or comorbid depression and anxiety stratified by age and sex.

## Introduction

Depression and anxiety are associated with an increased risk of multimorbidity and premature death from natural causes,^1,2,3^ and may be associated with a more rapid accumulation of chronic medical conditions commonly observed in older people, a proxy for accelerated aging.^4,5,6^ Although a direct link between depression, anxiety, and accelerated aging has not been demonstrated, there is increasing evidence of shared biological mechanisms between depression and chronic conditions known to occur later in life,^7^ as well as measures of cellular aging.^8^

Depression and anxiety disorders frequently cooccur,^9,10,11^ which is associated with greater illness severity and worse treatment outcomes than with either condition alone.^12,13,14,15^ Depression and anxiety are also associated with increased risk of comorbid general medical illnesses,^16,17^ suggesting that comorbid depression and anxiety may further increase the risk of multimorbidity from chronic illnesses beyond the risks associated with each individually.^18^ Previous longitudinal research has focused mainly on the associations between baseline depression or anxiety and the later occurrence of single chronic general medical conditions.^19,20,21,22,23,24,25,26,27,28,29,30^ Studies focused on depression or anxiety and the risk of multiple illnesses have been cross-sectional in nature^31,32,33,34,35^ or have considered longitudinal associations with individual conditions among those illnesses and not the risks or rates of acquiring cooccurring illnesses.^36,37,38,39,40^ Very few studies have directly compared the longitudinal risks of new chronic conditions or cooccurring conditions in people with depression, anxiety, and comorbid depression and anxiety within a single cohort,^41^ and to our knowledge, none has compared the rates of accumulation of chronic illnesses among these same groups of individuals. This is an important question, given the relatively early ages of onset of depression and anxiety and the possibility of a synergistic interaction of depression and anxiety in increasing the prevalence and costs of multimorbidity worldwide.^42,43^

Thus, we conducted a population-based retrospective cohort study to examine the associations of diagnosed depression, anxiety, and comorbid depression and anxiety with the accumulation of 15 common chronic conditions in 3 birthday age cohorts. We hypothesized that in all 3 cohorts the risks and rates of accumulation would be higher in cohorts with depression, anxiety, and comorbid depression and anxiety than in a reference cohort with neither depression nor anxiety and that the risk and rate of accumulation would be highest in individuals with comorbid depression and anxiety. We investigated these associations separately for women and men because of known sex differences in the occurrences of depression, anxiety, and several chronic conditions,^44,45,46^ and explored patterns of all-cause mortality for the main exposure groups within each birthday cohort.

## Methods

The Mayo Clinic and Olmsted Medical Center institutional review boards approved all research activities in this cohort study and waived informed consent, per Minnesota state privacy law, Statute §144.335.^47^ This study followed the Strengthening the Reporting of Observational Studies in Epidemiology (STROBE) reporting guideline for cohort studies.

### Data Source and Study Population

For this retrospective cohort study, the Rochester Epidemiology Project (REP) medical records-linkage system was used to identify residents of Olmsted County, Minnesota, in the 10-year period between January 1, 2005, and December 31, 2014. We excluded individuals who did not reach at least 1 anchoring birthday during the study period (<5%), and individuals who did not have any medical record with research authorization, per Minnesota legal requirements (<5%). The population enumeration obtained using the REP is similar to that obtained by the US Census, indicating that nearly the entire population of Olmsted County is captured by the system. Details about the REP have been previously reported.^48,49^

The sample was divided into cohorts anchored at birthday ages of 20, 40, and 60 years. The cohort aged 20 years included all individuals who celebrated their 20th birthday as Olmsted County residents between January 1, 2005, and December 31, 2014, and they were followed-up from the date of their 20th birthday (index date) through the end of the study, defined as the earliest of 3 dates: the date of death, the date of last medical contact with the REP, or December 31, 2017 (to allow for at least 3 years of follow-up after each of the anchoring birthdays). The cohorts aged 40 and 60 years were similarly defined, except that index dates occurred on the 40th or 60th birthday.

Individuals were excluded from our study if on the index birthday date they had evidence of diagnosed primary psychotic disorders (*International Classification of Diseases, Ninth Revision* [*ICD-9*]^50^ codes 295,V11.0, 297.x, 298.3, 298.4, 298.8, or 298.9), bipolar disorders (*ICD-9* codes 296.0x, 296.1, 296.4x, 296.5x, 296.6x, 296.7, or 296.8x), eating disorders (*ICD-9* codes 307.1 or 307.5x), dementia or delirium (*ICD-9* codes 290x, 293.0, 293.1, 294x, 310.0, 310.2, 310.8x, 310.9, 331.0, 331.1x, 331.2, 331.7, 331.7, 331.82, or 797), autism (*ICD-9* codes 299.00 or 299.01), or alcohol or other substance use disorders (*ICD-9* codes 291, 292, 303x, 304, 305x, or V113) based on the presence of at least 2 corresponding *ICD-9* diagnosis codes separated by more than 30 days in the 5 years before the index date (eFigure 1 in the Supplement). These conditions were excluded because of their associations with multimorbidity and mortality risk and because their presence may complicate the determination of depression and anxiety as exposures.^51,52,53,54,55,56^

### Main Exposure Groups

Main exposure groups in each age cohort were depression, anxiety, and comorbid depression and anxiety. Depression and anxiety were defined using *ICD-9* diagnostic codes^50^ for a unipolar depressive disorder or an anxiety disorder (eTable 1 in the Supplement). All index date birthdays were before the transition to *International Statistical Classification of Diseases and Related Health Problems, Tenth Revision* (*ICD-10)*^57^ codes for clinical practice on October 1, 2015; therefore, only *ICD-9* codes^50^ were used to define depression and anxiety. Persons met criteria for depression or anxiety if they received 2 or more diagnostic codes for the condition separated by more than 30 days in the 5 years before the index date. Individuals with depression alone met the definition for depression (but not anxiety), and individuals with anxiety alone met the definition for anxiety (but not depression). Individuals with comorbid depression and anxiety met the definitions for both depression and anxiety. Reference groups in each birthday age cohort included individuals who did not meet the definition for either depression or anxiety in the 5 years before the index date.

### Accumulation of Chronic Conditions

The primary outcome for this study was the accumulation of newly diagnosed chronic conditions. We considered 15 of 20 chronic conditions recommended by the US Department of Health and Human Services (eTable 2 in the Supplement).^58,59^ The modified list of chronic conditions excluded depression, schizophrenia, substance use disorders, dementia or delirium, and autism, given our focus on studying the associations of depression and anxiety with the accumulation of chronic conditions and because depression defined 2 of the exposure groups in this study. Chronic conditions were defined based on having 2 or more *ICD-9* or *ICD-10* diagnosis codes^50,57^ for a given condition separated by more than 30 days. This definition has been shown to result in a higher positive predictive value for identifying chronic conditions than relying on a single diagnosis code and is thus preferable for cohort studies.^60^ The date of onset of a given condition was defined as the date of the earliest diagnosis code that occurred more than 30 days after the first corresponding diagnosis code. According to this approach, acute events (eg, stroke) leading to death were analytically censored at the time of proximate death, consistent with this study’s focus on the accumulation of chronic conditions. Both *ICD-9* and *ICD-10* diagnosis codes^50,57^ were used to assess chronic conditions during follow-up (extending after October 1, 2015).

### All-Cause Mortality

All-cause mortality was an exploratory outcome in this study. Mortality was assessed by extracting REP electronic files, which contain detailed mortality information for residents of Olmsted County, including dates of death and causes of death as recorded on death certificates using *ICD-9* and *ICD-10* diagnosis codes.^48,50,57^

### Covariates

Age at cohort entry was fixed by design to index birthdays at ages 20, 40, and 60 years. Information on sex, self-reported race and ethnic group (Hispanic, non-Hispanic) as defined by the US Census Bureau, and self-reported education level were electronically extracted from REP indices for all cohort members. Race and ethnicity were included as covariates given the associations of both factors with the level of burden from chronic diseases and relative rates of chronic disease accumulation.^46,61^ Information on body mass index (BMI; calculated as weight in kilograms divided by height in meters squared) and smoking status at index date was electronically extracted from medical records.^48,49^

### Statistical Analysis

For each age cohort, the mean number of chronic conditions over time (years after the index birthday) was plotted by exposure group in men and women separately using Aalen-Johansen curves. To compare the risks of accumulating chronic conditions, hazard ratios (HRs) were estimated using Anderson-Gill regression models. Each event was treated equally; therefore, the analyses focused on time to the first event, then to the second event, and so forth. Adjusted HRs were calculated using inverse probability weights (IPWs) derived from generalized boosting models that included indicators for categories of covariates on which to balance for potential confounders. The covariates included for constructing balancing weights were race (in categories as Asian, Black, White, or other or unknown, including individuals who identified as Native American or Alaskan Native, who specifically indicated mixed race, or for whom no race information was available from any of their medical records), ethnicity (Hispanic or non-Hispanic), level of education (in categories as high school or less, some college, ≥4 years of college, and unknown), BMI (in categories as <25, 25 to <30, ≥30, and unknown), smoking status (never smoker, former smoker, current smoker, and unknown), and calendar year at index date (as an integer from 2005 through 2014).

The 15 chronic conditions were not excluded at index birthdays. Instead, all exposure groups within each birthday age cohort were balanced at index birthday on the listed covariates and on the prevalence of the 15 chronic conditions using IPWs. Using this approach, the Anderson-Gill regression models included only newly diagnosed (ie, incident) chronic conditions as outcomes. The balance across exposure groups obtained with IPWs was examined using the absolute value of the standardized difference of means,^62,63^ and a standardized difference of means less than 0.25 was defined as good balance of covariates. For sex-stratified analyses, IPWs were normalized so that the sum of the weights within a sex stratum equaled the number of individuals originally in that stratum. We tested for additive and multiplicative interactions between depression and anxiety with the risk of accumulating chronic conditions under the null hypothesis that no excess risk existed due to the interaction.^64,65^

To compare rates of accumulating new chronic conditions, we calculated the sex-specific mean annual incidence rate of accumulation of new chronic conditions per 100 person-years by exposure group and separately for each age cohort. The rates (and corresponding 95% CIs) were calculated by dividing the total number of newly acquired chronic conditions by the total number of person-years of follow-up in each cohort (and multiplying by 100). Excess accumulation of chronic conditions was calculated by subtracting pairwise incidence rates and constructing 95% CIs for the differences (eg, rates of accumulating new chronic conditions in individuals with comorbid depression and anxiety vs referent individuals).

For exploratory analyses, Kaplan-Meier survival curves were constructed with death from any cause as the outcome. Unweighted HRs and weighted HRs (with weights calculated as previously described) were calculated using Cox proportional hazard models. The proportionality assumption was tested visually and by including a time-dependent coefficient in the Cox models.

Analyses for the risks (HRs) of accumulation of chronic conditions were designed to assess relative differences between exposure groups. Analyses for the rates (incidence) of accumulation of chronic conditions were designed to assess absolute differences between exposure groups. All analyses were conducted using SAS statistical software version 9.4 (SAS Institute) and using R statistical software version 3.6.2 (R Project for Statistical Computing). All statistical tests of significance were undertaken at the conventional 2-tailed level of α = .05.

## Results

### Demographic and Clinical Characteristics

Among 40 360 individuals included across all 3 age cohorts, 21 516 (53.3%) were women. According to self report, 1830 individuals (4.5%) were Asian, 1696 individuals (4.2%) were Black, and 34 840 individuals (86.3%) were White. The study cohorts included 14 810 people in the cohort aged 20 years, 13 060 people in the cohort aged 40 years, and 12 490 people in the cohort aged 60 years (eFigure 1 in the Supplement). In the cohort aged 20 years, 1346 individuals (9.1%) had depression alone, 474 individuals (3.2%) had anxiety alone, and 512 individuals (3.5%) had comorbid depression and anxiety on the index date. Similar frequencies of depression, anxiety, and comorbid depression-anxiety were observed in the cohorts aged 40 or 60 years (Table 1). Exposure groups were not highly imbalanced before IPW (eFigures 2 in the Supplement); however, the use of IPW improved the balance of baseline characteristics across exposure groups, particularly for the comorbid depression and anxiety exposure group (standardized differences of means for nearly all variables were <0.25).

**Table 1.** Sociodemographic and Clinical Characteristics of Individuals With Depressive Disorders and/or Anxiety Disorders, Separately Within 3 Age Cohorts^a^

Characteristic	Birthday cohort, No. (%)												
	Age 20 y (n = 14 810)				Age 40 y (n = 13 060)				Age 60 y (n = 12 490)			
	Referent (n = 12 478)	Depression alone (n = 1346)	Anxiety alone (n = 474)	DA (n = 512)	Referent (n = 10 495)	Depression alone (n = 1443)	Anxiety alone (n = 600)	DA (n = 522)	Referent (n = 10 429)	Depression alone (n = 1328)	Anxiety alone (n = 334)	DA (N = 399)
Sex												
Women	6211 (49.8)	856 (63.6)	260 (54.9)	371 (72.5)	5356 (51.0)	1036 (71.8)	361 (60.2)	377 (72.2)	5279 (50.6)	901 (67.8)	221 (66.2)	287 (71.9)
Men	6267 (50.2)	490 (36.4)	214 (45.1)	141 (27.5)	5139 (49.0)	407 (28.2)	239 (39.8)	145 (27.8)	5150 (49.4)	427 (32.2)	113 (33.8)	112 (28.1)
Follow-up, median (IQR), y	5.9 (3.8-8.7)	6.5 (4.2-9.2)	5.3 (3.7-7.7)	5.5 (3.9-8.4)	7.5 (5.0-10.2)	7.1 (4.6-9.7)	6.7 (4.6-9.1)	6.3 (4.1-8.8)	6.9 (4.7-9.7)	6.6 (4.5-9.4)	7.0 (4.7-9.6)	6.3 (4.3-9.3)
Race												
Asian	573 (4.6)	29 (2.2)	15 (3.2)	15 (2.9)	719 (6.9)	36 (2.5)	21 (3.5)	7 (1.3)	358 (3.4)	40 (3.0)	8 (2.4)	9 (2.3)
Black	833 (6.7)	45 (3.3)	13 (2.7)	19 (3.7)	511 (4.9)	33 (2.3)	13 (2.2)	18 (3.4)	180 (1.7)	17 (1.3)	2 (0.6)	12 (3.0)
White	10 341 (82.9)	1185 (88.0)	419 (88.4)	448 (87.5)	8649 (82.4)	1292 (89.5)	530 (88.3)	469 (89.8)	9593 (92.0)	1234 (92.9)	313 (93.7)	367 (92.0)
Other or unknown^b^	731 (5.9)	87 (6.5)	27 (5.7)	30 (5.9)	616 (5.9)	82 (5.7)	36 (6.0)	28 (5.4)	298 (2.9)	37 (2.8)	11 (3.3)	11 (2.8)
Ethnicity												
Hispanic	632 (5.1)	78 (5.8)	26 (5.5)	28 (5.5)	594 (5.7)	81 (5.6)	29 (4.8)	30 (5.7)	350 (3.4)	51 (3.8)	7 (2.1)	16 (4.0)
Non-Hispanic	11 846 (94.9)	1268 (94.2)	448 (94.5)	484 (94.5)	9901 (94.3)	1362 (94.4)	571 (95.2)	492 (94.3)	10 079 (96.6)	1277 (96.2)	327 (97.9)	383 (96.0)
Education level												
≤High school	2390 (19.2)	337 (25.0)	100 (21.1)	121 (23.6)	1327 (12.6)	157 (10.9)	64 (10.7)	64 (12.3)	1978 (19.0)	260 (19.6)	64 (19.2)	85 (21.3)
Some college	3578 (28.7)	482 (35.8)	158 (33.3)	198 (38.7)	2668 (25.4)	483 (33.5)	182 (30.3)	176 (33.7)	3157 (30.3)	432 (32.5)	124 (37.1)	134 (33.6)
≥4 y college	3071 (24.6)	258 (19.2)	123 (25.9)	108 (21.1)	5121 (48.8)	660 (45.7)	287 (47.8)	237 (45.4)	4498 (43.1)	570 (42.9)	123 (36.8)	155 (38.8)
Unknown	3439 (27.6)	269 (20.0)	93 (19.6)	85 (16.6)	1379 (13.1)	143 (9.9)	67 (11.2)	45 (8.6)	796 (7.6)	66 (5.0)	23 (6.9)	25 (6.3)
Smoking status												
Never	6263 (50.2)	523 (38.9)	250 (52.7)	214 (41.8)	4830 (46.0)	561 (38.9)	273 (45.5)	200 (38.3)	4079 (39.1)	446 (33.6)	128 (38.3)	124 (31.1)
Former	633 (5.1)	152 (11.3)	41 (8.6)	68 (13.3)	1987 (18.9)	376 (26.1)	137 (22.8)	156 (29.9)	3673 (35.2)	551 (41.5)	117 (35.0)	164 (41.1)
Current	983 (7.9)	267 (19.8)	47 (9.9)	105 (20.5)	1326 (12.6)	267 (18.5)	96 (16.0)	98 (18.8)	1170 (11.2)	188 (14.2)	35 (10.5)	64 (16.0)
Unknown	4599 (36.9)	404 (30.0)	136 (28.7)	125 (24.4)	2352 (22.4)	239 (16.6)	94 (15.7)	68 (13.0)	1507 (14.5)	143 (10.8)	54 (16.2)	47 (11.8)
BMI												
<25	8653 (69.3)	874 (64.9)	339 (71.5)	323 (63.1)	3317 (31.6)	378 (26.2)	231 (38.5)	125 (23.9)	2336 (22.4)	267 (20.1)	97 (29.0)	88 (22.1)
25 to <30	1803 (14.4)	213 (15.8)	69 (14.6)	79 (15.4)	3560 (33.9)	399 (27.7)	191 (31.8)	144 (27.6)	3794 (36.4)	409 (30.8)	127 (38.0)	116 (29.1)
≥30	1475 (11.8)	242 (18.0)	59 (12.4)	104 (20.3)	3329 (31.7)	655 (45.4)	175 (29.2)	248 (47.5)	4185 (40.1)	647 (48.7)	109 (32.6)	193 (48.4)
Unknown	547 (4.4)	17 (1.3)	7 (1.5)	6 (1.2)	289 (2.8)	11 (0.8)	3 (0.5)	5 (1.0)	114 (1.1)	5 (0.4)	1 (0.3)	2 (0.5)
Other conditions												
Hypertension	51 (0.4)	5 (0.4)	4 (0.8)	2 (0.4)	710 (6.8)	149 (10.3)	67 (11.2)	59 (11.3)	3690 (35.4)	554 (41.7)	129 (38.6)	178 (44.6)
CHF	2 (<0.1)	0	1 (0.2)	0	8 (0.1)	1 (0.1)	4 (0.7)	2 (0.4)	98 (0.9)	17 (1.3)	2 (0.6)	10 (2.5)
CAD	1 (<0.1)	1 (0.1)	1 (0.2)	0	37 (0.4)	9 (0.6)	2 (0.3)	5 (1.0)	775 (7.4)	112 (8.4)	16 (4.8)	38 (9.5)
Cardiac arrhythmias	161 (1.3)	27 (2.0)	12 (2.5)	28 (5.5)	314 (3.0)	41 (2.8)	59 (9.8)	56 (10.7)	726 (7.0)	141 (10.6)	45 (13.5)	61 (15.3)
Hyperlipidemia	76 (0.6)	13 (1.0)	2 (0.4)	2 (0.4)	1167 (11.1)	219 (15.2)	76 (12.7)	92 (17.6)	4850 (46.5)	723 (54.4)	178 (53.3)	240 (60.2)
Stroke	6 (<0.1)	0	0	1 (0.2)	21 (0.2)	9 (0.6)	2 (0.3)	3 (0.6)	113 (1.1)	30 (2.3)	6 (1.8)	12 (3.0)
Arthritis	65 (0.5)	5 (0.4)	4 (0.8)	3 (0.6)	331 (3.2)	79 (5.5)	27 (4.5)	43 (8.2)	2029 (19.5)	414 (31.2)	72 (21.6)	140 (35.1)
Asthma	1203 (9.6)	144 (10.7)	50 (10.5)	80 (15.6)	529 (5.0)	126 (8.7)	21 (3.5)	66 (12.6)	478 (4.6)	115 (8.7)	14 (4.2)	46 (11.5)
Cancer^c^	46 (0.4)	4 (0.3)	3 (0.6)	1 (0.2)	222 (2.1)	37 (2.6)	14 (2.3)	16 (3.1)	819 (7.9)	129 (9.7)	34 (10.2)	44 (11.0)
CKD	35 (0.3)	4 (0.3)	3 (0.6)	0	97 (0.9)	21 (1.5)	6 (1.0)	12 (2.3)	246 (2.4)	52 (3.9)	7 (2.1)	19 (4.8)
COPD	113 (0.9)	23 (1.7)	3 (0.6)	12 (2.3)	149 (1.4)	35 (2.4)	10 (1.7)	20 (3.8)	312 (3.0)	83 (6.3)	10 (3.0)	34 (8.5)
Diabetes	89 (0.7)	19 (1.4)	3 (0.6)	3 (0.6)	512 (4.9)	114 (7.9)	25 (4.2)	53 (10.2)	2252 (21.6)	373 (28.1)	61 (18.3)	124 (31.1)
Hepatitis	24 (0.2)	6 (0.4)	1 (0.2)	0	76 (0.7)	7 (0.5)	1 (0.2)	0	68 (0.7)	24 (1.8)	2 (0.6)	7 (1.8)
HIV infection	2 (<0.1)	1 (0.1)	0	0	8 (0.1)	0	0	3 (0.6)	3 (<0.1)	0	1 (0.3)	0
Osteoporosis	3 (<0.1)	0	0	1 (0.2)	28 (0.3)	2 (0.1)	1 (0.2)	3 (0.6)	308 (3.0)	64 (4.8)	12 (3.6)	29 (7.3)

Abbreviations: BMI, body mass index (calculated as weight in kilograms divided by height in meters squared); CAD, coronary artery disease; CHF, congestive heart failure; CKD, chronic kidney disease; COPD, chronic obstructive pulmonary disease; DA, comorbid depression and anxiety.

^a^ Individuals were excluded at index birthday if they had autism, dementia or delirium, schizophrenia or psychoses, or substance abuse disorders. In addition, individuals with a depressive disorder in the context of a bipolar disorder and individuals with an eating disorder were excluded at index date.

^b^ Includes individuals who identified as Native American or Alaskan Native, who specifically indicated mixed race, or for whom no race information was available from any of their medical records.

^c^ Cancer includes all types of cancer except nonmelanoma skin cancer.

### Multimorbidity Accumulation in Women

**Figure 1.**
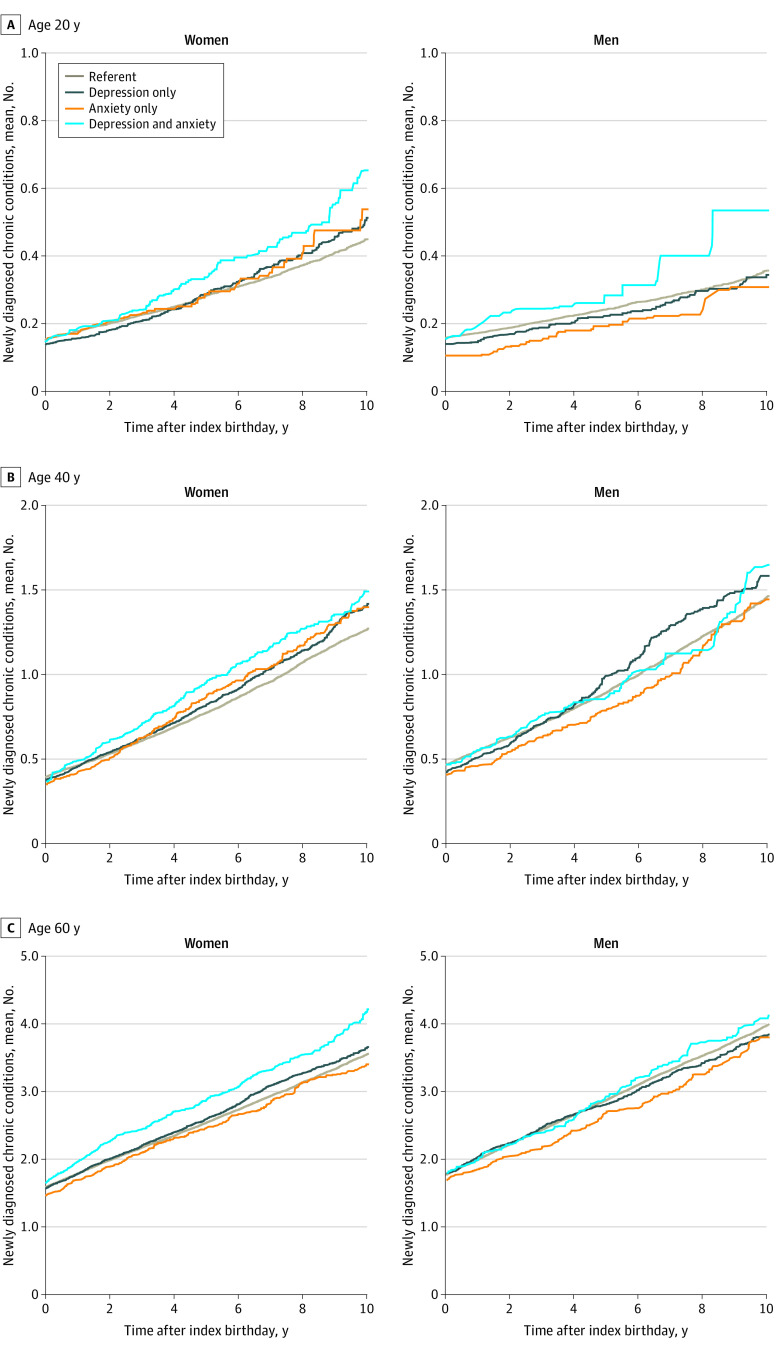
Accumulation of Chronic Conditions Over Time in Women and Men With Depression, Anxiety, Comorbid Depression and Anxiety, and Neither Depression nor Anxiety (Referent Group)

Estimates for risk of accumulating chronic conditions in each birthday age cohort are summarized in Table 2 and comparative rates of multimorbidity accumulation are summarized in Table 3. In both unweighted and weighted models and in all 3 cohorts, the risk of accumulating chronic conditions was significantly increased in women with depression (cohort aged 20 years: weighted HR, 1.20 [95% CI, 1.02-1.42]; cohort aged 40 years: weighted HR, 1.20 [95% CI, 1.10-1.31]; cohort aged 60 years: weighted HR, 1.09 [95% CI, 1.02-1.16]) and women with comorbid depression and anxiety (cohort aged 20 years: weighted HR, 1.60 [95% CI, 1.28-1.99]; cohort aged 40 years: weighted HR, 1.41 [95% CI, 1.21-1.65]; cohort aged 60 years: weighted HR, 1.29 [95% CI, 1.15-1.44]) compared with women with neither depression nor anxiety. For women in the cohort aged 40 years, anxiety was also associated with higher risk compared with referent women (weighted HR, 1.24; 95% CI, 1.08-1.43) (Table 2; Figure 1).

**Table 2.** Accumulation of Chronic Conditions Stratified by Age Cohort and Sex

Birthday cohort	No.			Unadjusted analyses		Adjusted analyses^a^		Interactions, *P* value	
	Individuals	Persons-years	Events^b^	HR (95% CI)^c^	*P* value	HR (95% CI)^c^	*P* value	M^d^	Additive^e^
**Women^f^**									
Age 20 y									
Referent	6211	40 482	1121	1 [Reference]	NA	1 [Reference]	NA	NA	NA
Depression alone	856	5839	242	1.49 (1.29-1.71)	<.001	1.20 (1.02-1.42)	.03	NA	NA
Anxiety alone	260	1589	56	1.31 (1.00-1.70)	.048	1.24 (0.94-1.64)	.13	NA	NA
Comorbid depression and anxiety	371	2391	121	1.84 (1.52-2.23)	<.001	1.60 (1.28-1.99)	<.001	.69	.50
Age 40 y									
Referent	5356	40 955	3370	1 [Reference]	NA	1 [Reference]	NA	NA	NA
Depression alone	1036	7615	875	1.41 (1.30-1.52)	<.001	1.20 (1.10-1.31)	<.001	NA	NA
Anxiety alone	361	2579	264	1.26 (1.10-1.44)	.001	1.24 (1.08-1.43)	.002	NA	NA
Comorbid depression and anxiety	377	2521	335	1.65 (1.46-1.86)	<.001	1.41 (1.21-1.65)	<.001	.55	.80
Age 60 y									
Referent	5279	38 557	7566	1 [Reference]	NA	1 [Reference]	NA	NA	NA
Depression alone	901	6311	1407	1.14 (1.07-1.21)	<.001	1.09 (1.02-1.16)	.01	NA	NA
Anxiety alone	221	1592	306	0.98 (0.87-1.10)	.74	1.05 (0.92-1.20)	.44	NA	NA
Comorbid depression and anxiety	287	1941	511	1.35 (1.22-1.48)	<.001	1.29 (1.15-1.44)	<.001	.12	.09
**Men^f^**									
Age 20 y									
Referent	6267	38 094	709	1 [Reference]	NA	1 [Reference]	NA	NA	NA
Depression alone	490	3187	86	1.44 (1.15-1.80)	.002	1.06 (0.81-1.37)	.68	NA	NA
Anxiety alone	214	1215	31	1.41 (0.98-2.02)	.07	1.13 (0.76-1.67)	.56	NA	NA
Comorbid depression and anxiety	141	801	26	1.78 (1.19-2.66)	.005	1.77 (1.08-2.91)	.02	.20	.18
Age 40 y									
Referent	5139	37 784	3634	1 [Reference]	NA	1 [Reference]	NA	NA	NA
Depression disorder alone	407	2823	359	1.34 (1.19-1.51)	<.001	1.21 (1.06-1.39)	.005	NA	NA
Anxiety alone	239	1618	161	1.05 (0.90-1.24)	.53	1.03 (0.86-1.23)	.75	NA	NA
Comorbid depression and anxiety	145	935	107	1.21 (0.99-1.48)	.06	1.07 (0.84-1.36)	.58	.25	.24
Age 60 y									
Referent	5150	36 466	8326	1 [Reference]	NA	1 [Reference]	NA	NA	NA
Depression alone	427	2954	687	1.02 (0.94-1.10)	.67	0.96 (0.88-1.05)	.35	NA	NA
Anxiety alone	113	804	165	0.90 (0.77-1.05)	.17	0.92 (0.78-1.07)	.27	NA	NA
Comorbid depression and anxiety	112	762	196	1.13 (0.97-1.30)	.11	1.07 (0.91-1.26)	.44	.09	.09

Abbreviations: HR, hazard ratio; M, multiplicative.

^a^ Adjusted models are weighted using weights developed from generalized boosting models to balance the 4 exposure groups at index birthday on potential confounders, including calendar year of birthday, race, ethnicity, education, smoking status, body mass index, and prevalence of each of the 15 chronic conditions.

^b^ Events for accumulation of additional chronic conditions included 15 chronic conditions: hypertension, congestive heart failure, coronary artery disease, cardiac arrhythmias, hyperlipidemia, stroke, arthritis, asthma, cancer, chronic kidney disease, chronic obstructive pulmonary disease, diabetes, hepatitis, HIV infection, and osteoporosis.

^c^ Time-to-event HRs are estimated from Andersen-Gill models.

^d^ The M interaction test is given by including the term (depression × anxiety) in the regression model.

^e^ The additive interaction test *P* value is calculated from the relative excess risk due to interaction method.^65^

^f^ Formal tests of sex-interaction were performed for adjusted analyses to determine whether the profile of HRs in women differed from those in men. The global test of sex-interaction simultaneously tests whether the HRs for depression alone, anxiety alone, and comorbid depression and anxiety differed between men and women. The global sex-interaction test was not statistically significant for the age 20 years cohorts (*P* = .78); however, the global sex-interaction test was statistically significant in the age 40 years (*P* = .02) and age 60 years (*P* = .008) cohorts.

**Table 3.** Mean Annual Rate of Accumulation of New Conditions Stratified by Age Cohort and Sex

Birthday cohort	Group, rate of new chronic conditions per 100 person-years (95% CI)			
	Reference	Depression alone	Anxiety alone	Comorbid depression and anxiety
Women				
Age 20 y	2.84 (2.68-3.00)	3.40 (2.93-3.88)	3.51 (2.64-4.38)	4.58 (3.77-5.38)
Age 40 y	8.49 (8.22-8.76)	10.2 (9.49-10.8)	10.6 (9.43-11.7)	12.0 (10.8-13.2)
Age 60 y	19.7 (19.3-20.1)	21.4 (20.4-22.4)	20.7 (18.7-22.7)	25.3 (23.5-27.2)
Men				
Age 20 y	1.89 (1.76-2.03)	2.03 (1.54-2.52)	2.10 (1.30-2.89)	3.25 (2.01-4.50)
Age 40 y	9.69 (9.39-9.99)	11.7 (10.6-12.9)	9.98 (8.59-11.4)	10.4 (8.55-12.2)
Age 60 y	22.8 (22.4-23.3)	21.9 (20.4-23.4)	20.9 (18.1-23.7)	24.4 (21.3-27.5)

**Figure 2.**
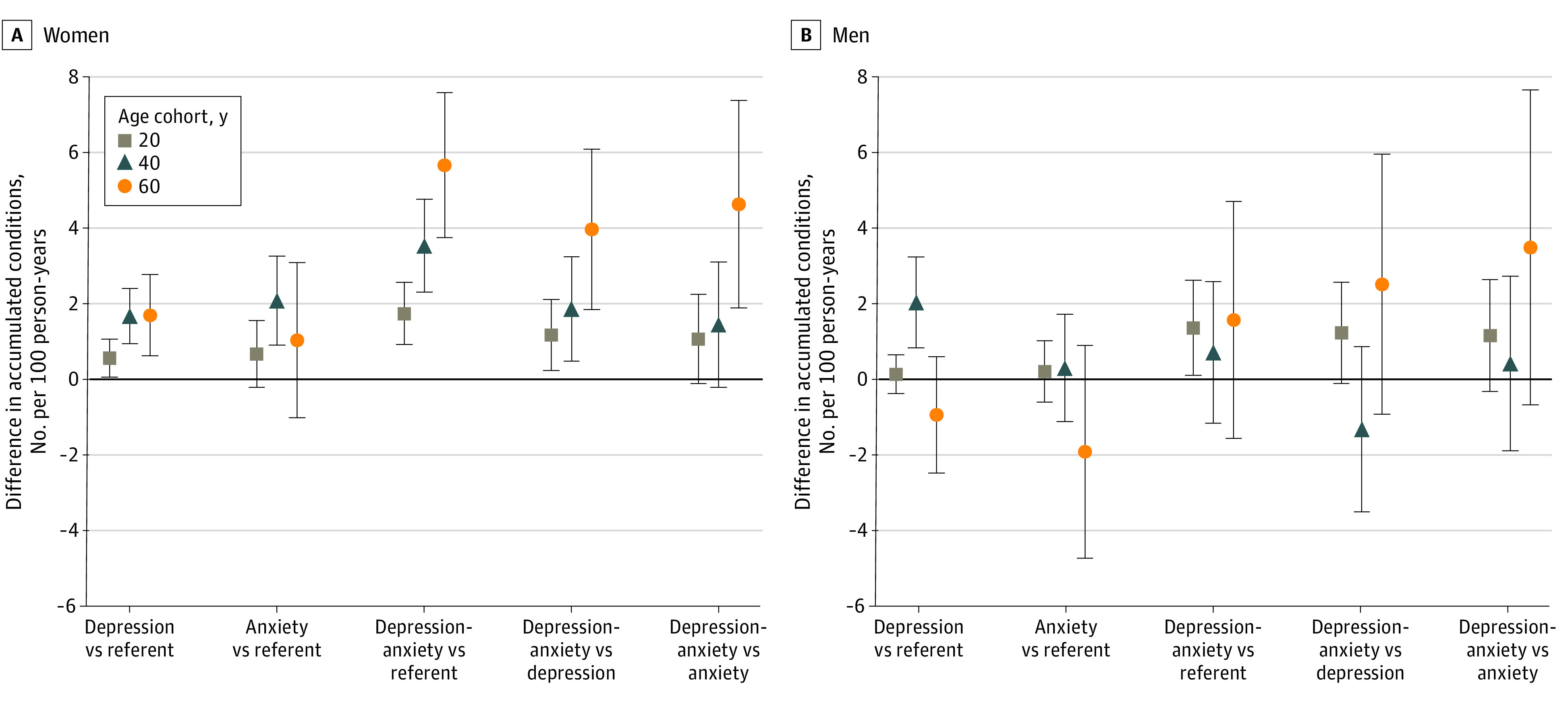
Pairwise Comparisons Between Exposure Groups of the Mean Annual Rate of Accumulation of New Conditions per 100 Person-Years in 3 Birthday Age Cohorts by Sex Referent refers to individuals in the same age cohort without depression or anxiety. Whiskers indicate 95% CIs.

For women, the rates of accumulation of chronic conditions were significantly higher in all 3 birthday cohorts for the comorbid depression and anxiety group than the depression group and reference group and higher than the anxiety group for the cohort aged 60 years (Table 3; Figure 2). The differences in rates of accumulation of chronic conditions were significantly higher for women with depression than women in the reference group in all 3 birthday cohorts (cohort aged 20 years: 0.6 [95% CI, 0.1-1.1] per 100 person-years; cohort aged 40 years: 1.7 [95% CI, 0.9-2.4] per 100 person-years; cohort aged 60 years: 1.7 [95% CI, 0.6-2.8] per 100 person-years), and for women with anxiety compared with the reference group in only the cohort aged 40 years (2.1 [95% CI, 0.9-3.3] per 100 person-years) (Figure 2). Women with comorbid depression and anxiety accumulated from 1.7 (95% CI, 0.9-2.6) more comorbid conditions per 100 person-years in the cohort aged 20 years to 5.7 (95% CI, 3.7-7.6) more comorbid conditions per 100 person-years in the cohort aged 60 years than women in the reference groups. Women with comorbid depression and anxiety accumulated more chronic conditions per 100 person-years than women with depression alone in all age cohorts and more than women with anxiety alone only in the cohort aged 60 years (Figure 2).

### Multimorbidity Accumulation in Men

For men, there was a significantly higher risk for accumulating chronic conditions with depression and comorbid depression and anxiety compared with men in the reference group in the cohort aged 20 years in unweighted models; however, the increased risk remained significant only for comorbid depression and anxiety in weighted models (HR, 1.77 [95% CI, 1.08-2.91]) (Table 2; Figure 1). The risk of accumulating chronic conditions was also significantly increased with comorbid depression and anxiety compared with men in the reference group in the cohort aged 40 years, but only in unweighted models (Table 2).

Rates of accumulation of chronic conditions were highest for men with comorbid depression and anxiety in all birthday cohorts (Table 3). However, compared with men in the reference group, the differences in rates of accumulation of chronic conditions were significantly higher in men with comorbid depression and anxiety only in the cohort aged 20 years (difference, 1.4 [95% CI, 0.1-2.6] per 100 person-years) and for men with depression in only the cohort aged 40 years (difference, 2.0 [95% CI, 0.8-3.2] per 100 person-years) (Table 3; Figure 2).

### Statistical Tests for Interaction

Formal statistical tests for interaction were conducted to analyze the joint associations of depression and anxiety with the risk of accumulating chronic conditions. For women and men, statistical tests for additive or multiplicative interaction between depression and anxiety on the risk of accumulating chronic conditions were not statistically significant (Table 2).

### Exploratory Analyses: Mortality

The results of exploratory analyses of all-cause mortality risk are summarized in eTable 3 in the Supplement and are visually displayed in eFigure 3 in the Supplement for the cohorts aged 60 years only. For women, depression and comorbid depression and anxiety were significantly associated with all-cause mortality in the cohorts aged 40 and 60 years in unweighted models, but only depression was associated with all-cause mortality in both birthday cohorts in weighted models (eTable 3 in the Supplement). There was no significant association between anxiety and mortality for women in any of the cohorts.

For men, the risk of mortality was significantly increased in unweighted and weighted models for depression, but not comorbid depression and anxiety or anxiety alone, in the cohort aged 60 years (eTable 3 in the Supplement). The risk of mortality was not significantly increased for men in any exposure group in the cohorts aged 20 or 40 years.

## Discussion

To our knowledge, this cohort study is the first study comparing the longitudinal risk of accumulating chronic conditions in individuals with depression, anxiety, or comorbid depression and anxiety across the age spectrum within a geographically defined population. The risk of accumulating chronic conditions was significantly higher in women with depression and comorbid depression and anxiety than women in the reference group in each of 3 birthday cohorts (ages 20, 40, and 60 years), with similar observations for men in the cohort aged 20 years. Rates of accumulation of chronic conditions were also highest for women and men with comorbid depression and anxiety.

An extensive body of prior research has documented longitudinal associations of depression, anxiety, or depressive and anxious symptoms with the later onset or diagnoses of individual cardiovascular diseases,^19,24,25,26,27,28,30,66,67^ metabolic diseases,^19,21,22^ certain types of cancer,^20^ atopic illnesses,^23^ and chronic or recurring pain syndromes.^68,69^ More recent work, mainly cross-sectional or retrospective in nature, has focused on the risk of multiple chronic conditions, showing more chronic health conditions in people with depression and individuals with anxiety.^31,32,33,34,70^ Longitudinal studies of mood or anxiety disorders and the later occurrence of multiple chronic conditions are far fewer, but they also reported associations of depression or anxiety with individual conditions,^36,37,38^ including one of the largest studies to date, a 2020 study by Momen et al,^40^ that showed associations of mood disorders (including unipolar and bipolar depressive disorders) and neurotic disorders (including anxiety disorders, posttraumatic stress disorder [PTSD] and related conditions, obsessive-compulsive disorder, and somatoform disorders) with increases in incident general medical conditions within 9 broad categories, each considered individually. In that study,^40^ the cumulative incidence of each of the 9 groups of conditions was significantly higher in people with mood disorders and in people with neurotic disorders; however, comorbid depression and anxiety as an exposure group and the cumulative incidence of multiple conditions were not included. There are few studies of the cumulative burden of incident chronic illnesses in people with baseline depression or anxiety. The English Longitudinal Study of Ageing^71^ showed a higher incidence of general medical conditions and higher illness burden (based on the number of incident conditions) in people with baseline depression. Another longitudinal study from Australia^72^ documented a greater than 4-fold increase in the cumulative 20-year incidence of multiple chronic physical conditions in a cohort of women with depressive symptoms compared with a matched cohort of women without depression. Our results extend beyond prior research by documenting a more rapid rate of accumulation of newly diagnosed chronic conditions among women over time with depression and anxiety separately, and an even higher rate of accumulation when depression and anxiety cooccurred.

The identification of treatable risk factors for multimorbidity, such as depression and anxiety, is vital from a public health perspective because of the high prevalence and associated costs of multimorbidity,^42,43^ which are expected to continue to increase.^73^ Depression and anxiety are associated with incident general medical illnesses at a scale similar to obesity and smoking.^74^ To our knowledge, no previous studies have directly compared the longitudinal rates of accumulation of chronic illnesses among people with depression, anxiety, and comorbid depression and anxiety. This knowledge gap is important because approximately half of individuals with depression have a comorbid anxiety disorder, and vice-versa.^75,76^ A cross-sectional study documented a higher lifetime risk of various prevalent chronic conditions (mainly cardiovascular diseases and chronic pain) in people with comorbid depression and anxiety compared with individuals with either condition alone.^41^ Our study contributes longitudinal findings showing higher rates of accumulation of chronic diseases with comorbid depression and anxiety compared with neither depression nor anxiety or with depression alone across the age span in women and compared with neither depression nor anxiety in younger men.

This study focused on the associations between depression and anxiety with the accumulation of chronic conditions, a process that may be driven by age-related homeostatic dysregulation across multiple organ systems.^46,77^ Our findings suggest that common mechanisms may underly depression and anxiety as well as aging and that these mechanisms may be magnified when depression and anxiety cooccur. Multiple biological mechanisms are implicated in this process, including chronic inflammation, neuroendocrine dysregulation, oxidative stress, mitochondrial dysfunction, and others.^78,79,80,81,82^ Nearly all of these mechanisms are associated with psychosocial stress,^83^ thus providing an additional link between common stress-mediated psychiatric disorders, like depression and anxiety, and chronic somatic illnesses. From a translational viewpoint, the relative overexpression of specific biological mechanisms in individuals with depression or anxiety, activated or enhanced by the cumulative effects of stress, may be reflected in specific multimorbidity patterns that involve distinct groups of chronic somatic illnesses.

For women aged 40 or 60 years, the maximum pairwise differences in rates of accumulation of new conditions between comorbid depression and anxiety and depression and anxiety separately was 4.6 new conditions per 100 person-years. However, even this increase may be clinically significant. Prior research has shown that multimorbidity is a known risk factor for mortality,^6,84^ and the differences in accumulation of chronic conditions in our study may be large enough to contribute to the higher risk of all-cause mortality with comorbid depression and anxiety. Moreover, just 1 newly diagnosed chronic illness per 12 to 36 months is associated with increasing the risk of functional dependency by 2-fold, a known factor involved in the association between multimorbidity and mortality in older adults.^85^ Our findings support the need for managing comorbid depression and anxiety,^86^ which may help lower the risk of premature mortality associated with multimorbidity.

A 2018 study^87^ reported that depressive or anxiety symptom severity, rather than discrete diagnoses, is associated with multimorbidity development. Our focus on depression and anxiety disorders was intentional, because we used diagnostic codes for discrete depressive and anxiety disorders. Although depression with anxiety symptoms is a useful illness subtype,^88,89^ documented associations between cooccurring depressive and anxiety disorders and worse health and functioning provide clinical justification for our approach.^12,13,14,15^

The anxiety disorders in this study included PTSD and acute stress disorder (ASD), consistent with the definition of anxiety disorders in the Medical Expenditure Panel Survey.^90^ There were at least 3 reasons for including PTSD and ASD as anxiety disorders in this study. First, PTSD and ASD have been classified as trauma- and stressor-related disorders, separate from anxiety disorders, only since 2013.^91^ Therefore, during nearly the entire time in which cohort members were identified (January 1, 2005, to December 31, 2014), ASD and PTSD were considered anxiety disorders. Second, although the broader emotional experience of patients with PTSD and ASD extends beyond fear and anxiety,^92^ both are central constructs for the development of PTSD and ASD, and fear and avoidance are fundamental targets of treatment for both conditions.^93^ And finally, PTSD has been associated with increased risk for a variety of chronic health problems, including incident cardiometabolic diseases, even after adjusting for depression.^25,26,40^

The association between sex and multimorbidity has received limited investigation.^94,95^ This work highlights potentially important sex differences in the association between depression and anxiety and multimorbidity accumulation. Our study design was based on known sex dimorphisms in the prevalence of depression, anxiety, and several chronic diseases^44,45,46,96^ and was consistent with recent calls for additional studies on sex differences in somatic-mental multimorbidity to support precision medicine.^97,98^ We did not have access to data on self-reported gender identity, so we were unable to examine associations involving gender, separate from biological sex, on patterns of multimorbidity acquisition.

We found an increased risk of accumulating chronic conditions with depression and comorbid depression-anxiety in women across all age groups, but only in younger men. Although differences related to reporting of conditions, socioeconomic roles, and other social factors cannot be excluded, the susceptibility to increased risk of accumulating chronic conditions in women across the age span in our study may reflect heightened risk for stress- and trauma-related disorders, chronicity of anxiety symptoms, and cumulative outcomes associated with increased stress and inflammation in women, compared with men.^45,99,100^ In particular, potential sex differences in the sensitivity to outcomes associated with chronically increased stress-system activation and inflammation in the body may translate to not only higher risk of depression and anxiety,^101,102^ but also disproportionately increased accumulation of a wide range of stress-related chronic illnesses, including (but not limited to) cardiovascular diseases, metabolic disorders, obesity, certain cancers, atopic diseases, and pain syndromes, in women.^103,104,105^

The associations of depression and comorbid depression and anxiety with increased risk of accumulating chronic conditions isolated to only some age cohorts in men is more difficult to explain. The increasing prevalence of internalizing problems (characterized by depression and anxiety) impacting 21st century youth have been reported to be more pronounced in adolescent girls than boys.^106,107^ Such differences may reflect higher detection rates of mood and anxiety disorders among girls, consistent with the broader literature pointing to higher rates of undiagnosed mood and anxiety disorders in men relative to women.^108^ Improved mental health literacy and better recognition of male-typical symptoms of depression and anxiety may have resulted in reduced barriers to seeking or receiving treatment for mental health conditions among the men in the cohort aged 20 years relative to those in older cohorts. Such a difference may have resulted in the occurrence of more pairings of depression and/or anxiety with chronic condition in the medical records of younger men than older men in our study, a hypothesis that we were not able to test with this study design.

Our study had a number of strengths, including access to medical record data for all conditions of interest and for the full spectrum of primary and specialty care within a geographically defined population. The study cohorts included all age, sex, and racial and ethnicity groups, regardless of socioeconomic status, insurance status, and health care delivery setting.^49^ All 15 chronic health conditions in this research were chosen by the US Department of Health and Human Services as being high public health priorities for the nation and are among those suggested for international multimorbidity research.^109,110^ Additionally, the code sets used in this research were previously validated against medical record review of a representative sample of the underlying population.^60^

### Limitations

This study has some limitations. Mental health conditions and multimorbidity have a bidirectional association,^111^ and this work focused on only 1 direction. However, the study design was appropriate for assessing the longitudinal associations of depression and anxiety with the accumulation of chronic conditions. The most rapid accumulation of chronic conditions was consistently observed with comorbid depression and anxiety; however, significant additive or multiplicative synergistic interactions between depression and anxiety were not observed. The etiologic complexities of depression, anxiety, and the somatic conditions under consideration in this study may conform poorly to simple additive or multiplicative statistical associations. Our exposure and outcome definitions relied on data not collected for research purposes, and it is unclear how much the higher rates of chronic conditions among individuals with mental health conditions reflects differences in health care service utilization (resulting in accruing more diagnosis billing codes) vs greater incidence. Although the REP captures virtually the entire local Olmsted County, Minnesota, population compared with the US Census,^49^ some individuals with depression in the population may have been misclassified as being nondepressed, especially because diagnoses of depressive disorders may be missed in routine care settings. We did not include personality disorders in our definition of depression or anxiety, which is an important limitation, given that many personality disorders are difficult to differentiate from affective disorders, prevalent among individuals seeking health care services, and associated with higher morbidity and mortality compared with individuals without personality disorders.^112^ Our list of accumulated chronic conditions was limited to 15 conditions, and the actual dates of onset likely occurred before the estimated dates of onset using diagnosis codes. The risk of reverse causation bias (eg, indolent diseases increasing the risk of depression and anxiety) was minimized by the use of Anderson-Gill models that accounted only for new conditions but cannot be completely excluded, given the use of diagnosis codes to define the onset dates of conditions. Additionally, the generalizability of our results may be limited for populations more economically or racially diverse than our study cohort.

## Conclusions

In this cohort study of men and women from 3 birthday age cohorts, the risk of accumulating chronic conditions was increased in women with depression and comorbid depression-anxiety across the age span and in younger men. Compared with individuals without depression or anxiety, there was a higher rate of accumulation of comorbid conditions when depression and anxiety cooccurred among women across the age spectrum and among younger men.

## Notes

Supplement. **eTable 1.** List of the Diagnosis Codes Used to Define Depression and Anxiety **eTable 2.** List of Chronic Conditions and Corresponding Diagnosis Codes **eTable 3.** Risk of Death in Birthday Age Cohorts for Women and Men Separately **eFigure 1.** Inclusion of Individuals in Birthday Age Cohorts and Accumulation of Chronic Conditions **eFigure 2.** Balance of Characteristics by Birthday Age Cohort **eFigure 3.** Kaplan-Meier Estimates of Mortality in the Age 60 Years Cohort Separately for Women and Men

## References

[1] Ayuso-Mateos JL, Nuevo R, Verdes E, Naidoo N, Chatterji S. From depressive symptoms to depressive disorders: the relevance of thresholds. Br J Psychiatry. 2010;196(5):365-371. doi:10.1192/bjp.bp.109.071191 20435961

[2] Furukawa TA, Konno W, Morinobu S, Harai H, Kitamura T, Takahashi K. Course and outcome of depressive episodes: comparison between bipolar, unipolar and subthreshold depression. Psychiatry Res. 2000;96(3):211-220. doi:10.1016/S0165-1781(00)00212-2 11084217

[3] Meier SM, Mattheisen M, Mors O, Mortensen PB, Laursen TM, Penninx BW. Increased mortality among people with anxiety disorders: total population study. Br J Psychiatry. 2016;209(3):216-221. doi:10.1192/bjp.bp.115.171975 27388572 PMC5082973

[4] Fabbri E, An Y, Zoli M, . Association between accelerated multimorbidity and age-related cognitive decline in older Baltimore Longitudinal Study of Aging participants without dementia. J Am Geriatr Soc. 2016;64(5):965-972. doi:10.1111/jgs.14092 27131225 PMC4882249

[5] Kennedy BK, Berger SL, Brunet A, . Geroscience: linking aging to chronic disease. Cell. 2014;159(4):709-713. doi:10.1016/j.cell.2014.10.039 25417146 PMC4852871

[6] Rocca WA, Grossardt BR, Boyd CM, Chamberlain AM, Bobo WV, St Sauver JL. Multimorbidity, ageing and mortality: normative data and cohort study in an American population. BMJ Open. 2021;11(3):e042633. doi:10.1136/bmjopen-2020-042633 33741663 PMC7986688

[7] Wolkowitz OM, Reus VI, Mellon SH. Of sound mind and body: depression, disease, and accelerated aging. Dialogues Clin Neurosci. 2011;13(1):25-39. doi:10.31887/DCNS.2011.13.1/owolkowitz 21485744 PMC3181963

[8] Verhoeven JE, Révész D, Epel ES, Lin J, Wolkowitz OM, Penninx BW. Major depressive disorder and accelerated cellular aging: results from a large psychiatric cohort study. Mol Psychiatry. 2014;19(8):895-901. doi:10.1038/mp.2013.151 24217256

[9] Fawcett J, Kravitz HM. Anxiety syndromes and their relationship to depressive illness. J Clin Psychiatry. 1983;44(8 Pt 2):8-11. 6874657

[10] Rapaport MH. Prevalence, recognition, and treatment of comorbid depression and anxiety. J Clin Psychiatry. 2001;62(suppl 24):6-10. 11676431

[11] Regier DA, Boyd JH, Burke JD Jr, . One-month prevalence of mental disorders in the United States: based on five Epidemiologic Catchment Area sites. Arch Gen Psychiatry. 1988;45(11):977-986. doi:10.1001/archpsyc.1988.01800350011002 3263101

[12] Clayton PJ, Grove WM, Coryell W, Keller M, Hirschfeld R, Fawcett J. Follow-up and family study of anxious depression. Am J Psychiatry. 1991;148(11):1512-1517. doi:10.1176/ajp.148.11.1512 1928465

[13] Fawcett J, Scheftner WA, Fogg L, . Time-related predictors of suicide in major affective disorder. Am J Psychiatry. 1990;147(9):1189-1194. doi:10.1176/ajp.147.9.1189 2104515

[14] Fava M, Rush AJ, Alpert JE, . Difference in treatment outcome in outpatients with anxious versus nonanxious depression: a STAR*D report. Am J Psychiatry. 2008;165(3):342-351. doi:10.1176/appi.ajp.2007.06111868 18172020

[15] Vollrath M, Angst J. Outcome of panic and depression in a seven-year follow-up: results of the Zurich study. Acta Psychiatr Scand. 1989;80(6):591-596. doi:10.1111/j.1600-0447.1989.tb03031.x 2618782

[16] Bobo WV, Yawn BP, St Sauver JL, Grossardt BR, Boyd CM, Rocca WA. Prevalence of combined somatic and mental health multimorbidity: patterns by age, sex, and race/ethnicity. J Gerontol A Biol Sci Med Sci. 2016;71(11):1483-1491. doi:10.1093/gerona/glw032 26935110 PMC5055644

[17] Tully PJ, Harrison NJ, Cheung P, Cosh S. Anxiety and cardiovascular disease risk: a review. Curr Cardiol Rep. 2016;18(12):120. doi:10.1007/s11886-016-0800-3 27796859

[18] Scott KM, Bruffaerts R, Tsang A, . Depression-anxiety relationships with chronic physical conditions: results from the World Mental Health Surveys. J Affect Disord. 2007;103(1-3):113-120. doi:10.1016/j.jad.2007.01.015 17292480

[19] Birk JL, Kronish IM, Moise N, Falzon L, Yoon S, Davidson KW. Depression and multimorbidity: Considering temporal characteristics of the associations between depression and multiple chronic diseases. Health Psychol. 2019;38(9):802-811. doi:10.1037/hea0000737 31008648 PMC6706317

[20] Chida Y, Hamer M, Wardle J, Steptoe A. Do stress-related psychosocial factors contribute to cancer incidence and survival? Nat Clin Pract Oncol. 2008;5(8):466-475. doi:10.1038/ncponc1134 18493231

[21] Mezuk B, Eaton WW, Albrecht S, Golden SH. Depression and type 2 diabetes over the lifespan: a meta-analysis. Diabetes Care. 2008;31(12):2383-2390. doi:10.2337/dc08-0985 19033418 PMC2584200

[22] Rotella F, Mannucci E. Depression as a risk factor for diabetes: a meta-analysis of longitudinal studies. J Clin Psychiatry. 2013;74(1):31-37. doi:10.4088/JCP.12r07922 23419223

[23] Chida Y, Hamer M, Steptoe A. A bidirectional relationship between psychosocial factors and atopic disorders: a systematic review and meta-analysis. Psychosom Med. 2008;70(1):102-116. doi:10.1097/PSY.0b013e31815c1b71 18158379

[24] Smoller JW, Pollack MH, Wassertheil-Smoller S, . Panic attacks and risk of incident cardiovascular events among postmenopausal women in the Women’s Health Initiative Observational Study. Arch Gen Psychiatry. 2007;64(10):1153-1160. doi:10.1001/archpsyc.64.10.1153 17909127

[25] Kubzansky LD, Koenen KC, Spiro A III, Vokonas PS, Sparrow D. Prospective study of posttraumatic stress disorder symptoms and coronary heart disease in the Normative Aging Study. Arch Gen Psychiatry. 2007;64(1):109-116. doi:10.1001/archpsyc.64.1.109 17199060

[26] Roy SS, Foraker RE, Girton RA, Mansfield AJ. Posttraumatic stress disorder and incident heart failure among a community-based sample of US veterans. Am J Public Health. 2015;105(4):757-763. doi:10.2105/AJPH.2014.302342 25713943 PMC4358172

[27] Weissman MM, Markowitz JS, Ouellette R, Greenwald S, Kahn JP. Panic disorder and cardiovascular/cerebrovascular problems: results from a community survey. Am J Psychiatry. 1990;147(11):1504-1508. doi:10.1176/ajp.147.11.1504 2221163

[28] Kawachi I, Sparrow D, Vokonas PS, Weiss ST. Symptoms of anxiety and risk of coronary heart disease. the Normative Aging Study. Circulation. 1994;90(5):2225-2229. doi:10.1161/01.CIR.90.5.2225 7955177

[29] Shen B-J, Avivi YE, Todaro JF, . Anxiety characteristics independently and prospectively predict myocardial infarction in men the unique contribution of anxiety among psychologic factors. J Am Coll Cardiol. 2008;51(2):113-119. doi:10.1016/j.jacc.2007.09.033 18191733

[30] Albert CM, Chae CU, Rexrode KM, Manson JE, Kawachi I. Phobic anxiety and risk of coronary heart disease and sudden cardiac death among women. Circulation. 2005;111(4):480-487. doi:10.1161/01.CIR.0000153813.64165.5D 15687137

[31] Pruchno RA, Wilson-Genderson M, Heid AR. Multiple chronic condition combinations and depression in community-dwelling older adults. J Gerontol A Biol Sci Med Sci. 2016;71(7):910-915. doi:10.1093/gerona/glw025 26933159

[32] Smith DJ, Court H, McLean G, . Depression and multimorbidity: a cross-sectional study of 1,751,841 patients in primary care. J Clin Psychiatry. 2014;75(11):1202-1208. doi:10.4088/JCP.14m09147 25470083

[33] Glaesmer H, Brähler E, Gündel H, Riedel-Heller SG. The association of traumatic experiences and posttraumatic stress disorder with physical morbidity in old age: a German population-based study. Psychosom Med. 2011;73(5):401-406. doi:10.1097/PSY.0b013e31821b47e8 21636658

[34] Spitzer C, Barnow S, Völzke H, John U, Freyberger HJ, Grabe HJ. Trauma, posttraumatic stress disorder, and physical illness: findings from the general population. Psychosom Med. 2009;71(9):1012-1017. doi:10.1097/PSY.0b013e3181bc76b5 19834051

[35] Scott KM, Von Korff M, Angermeyer MC, . Association of childhood adversities and early-onset mental disorders with adult-onset chronic physical conditions. Arch Gen Psychiatry. 2011;68(8):838-844. doi:10.1001/archgenpsychiatry.2011.77 21810647 PMC3402030

[36] Holahan CJ, Pahl SA, Cronkite RC, Holahan CK, North RJ, Moos RH. Depression and vulnerability to incident physical illness across 10 years. J Affect Disord. 2010;123(1-3):222-229. doi:10.1016/j.jad.2009.10.006 19880190

[37] Patten SB, Williams JVA, Lavorato DH, Modgill G, Jetté N, Eliasziw M. Major depression as a risk factor for chronic disease incidence: longitudinal analyses in a general population cohort. Gen Hosp Psychiatry. 2008;30(5):407-413. doi:10.1016/j.genhosppsych.2008.05.001 18774423

[38] Poole L, Jackowska M. The association between depressive and sleep symptoms for predicting incident disease onset after 6-year follow-up: findings from the English Longitudinal Study of Ageing. Psychol Med. 2019;49(4):607-616. doi:10.1017/S0033291718001290 29807551 PMC6378411

[39] Chen C-M, Lee I-C, Su Y-Y, Mullan J, Chiu HC. The longitudinal relationship between mental health disorders and chronic disease for older adults: a population-based study. Int J Geriatr Psychiatry. 2017;32(9):1017-1026. doi:10.1002/gps.4561 27546556

[40] Momen NC, Plana-Ripoll O, Agerbo E, . Association between mental disorders and subsequent medical conditions. N Engl J Med. 2020;382(18):1721-1731. doi:10.1056/NEJMoa1915784 32348643 PMC7261506

[41] Scherrer JF, Chrusciel T, Zeringue A, . Anxiety disorders increase risk for incident myocardial infarction in depressed and nondepressed Veterans Administration patients. Am Heart J. 2010;159(5):772-779. doi:10.1016/j.ahj.2010.02.033 20435185

[42] Lehnert T, Heider D, Leicht H, . Review: health care utilization and costs of elderly persons with multiple chronic conditions. Med Care Res Rev. 2011;68(4):387-420. doi:10.1177/1077558711399580 21813576

[43] McPhail SM. Multimorbidity in chronic disease: impact on health care resources and costs. Risk Manag Healthc Policy. 2016;9:143-156. doi:10.2147/RMHP.S97248 27462182 PMC4939994

[44] Kessler RC. Epidemiology of women and depression. J Affect Disord. 2003;74(1):5-13. doi:10.1016/S0165-0327(02)00426-3 12646294

[45] McLean CP, Asnaani A, Litz BT, Hofmann SG. Gender differences in anxiety disorders: prevalence, course of illness, comorbidity and burden of illness. J Psychiatr Res. 2011;45(8):1027-1035. doi:10.1016/j.jpsychires.2011.03.006 21439576 PMC3135672

[46] St Sauver JL, Boyd CM, Grossardt BR, . Risk of developing multimorbidity across all ages in an historical cohort study: differences by sex and ethnicity. BMJ Open. 2015;5(2):e006413. doi:10.1136/bmjopen-2014-006413 25649210 PMC4322195

[47] Minn Stat. §144.335 (2006). Accessed March 31, 2022. https://www.revisor.mn.gov/statutes/2006/cite/144.335

[48] Rocca WA, Grossardt BR, Brue SM, . Data resource profile: expansion of the Rochester Epidemiology Project medical records-linkage system (E-REP). Int J Epidemiol. 2018;47(2):368-368j. doi:10.1093/ije/dyx268 29346555 PMC5913632

[49] St Sauver JL, Grossardt BR, Yawn BP, . Data resource profile: the Rochester Epidemiology Project (REP) medical records-linkage system. Int J Epidemiol. 2012;41(6):1614-1624. doi:10.1093/ije/dys195 23159830 PMC3535751

[50] World Health Organization. International Classification of Diseases, Ninth Revision (ICD-9). World Health Organization; 1977.

[51] Baxter AJ, Brugha TS, Erskine HE, Scheurer RW, Vos T, Scott JG. The epidemiology and global burden of autism spectrum disorders. Psychol Med. 2015;45(3):601-613. doi:10.1017/S003329171400172X 25108395

[52] Berkman ND, Lohr KN, Bulik CM. Outcomes of eating disorders: a systematic review of the literature. Int J Eat Disord. 2007;40(4):293-309. doi:10.1002/eat.20369 17370291

[53] Glei DA, Preston SH. Estimating the impact of drug use on US mortality, 1999-2016. PLoS One. 2020;15(1):e0226732. doi:10.1371/journal.pone.0226732 31940370 PMC6961845

[54] Laursen TM. Life expectancy among persons with schizophrenia or bipolar affective disorder. Schizophr Res. 2011;131(1-3):101-104. doi:10.1016/j.schres.2011.06.008 21741216

[55] Roshanaei-Moghaddam B, Katon W. Premature mortality from general medical illnesses among persons with bipolar disorder: a review. Psychiatr Serv. 2009;60(2):147-156. doi:10.1176/ps.2009.60.2.147 19176408

[56] Suvarna B, Suvarna A, Phillips R, Juster RP, McDermott B, Sarnyai Z. Health risk behaviours and allostatic load: a systematic review. Neurosci Biobehav Rev. 2020;108:694-711. doi:10.1016/j.neubiorev.2019.12.020 31846655

[57] World Health Organization. International Statistical Classification of Diseases, Tenth Revision (ICD-10). World Health Organization; 1992.

[58] Goodman RA, Posner SF, Huang ES, Parekh AK, Koh HK. Defining and measuring chronic conditions: imperatives for research, policy, program, and practice. Prev Chronic Dis. 2013;10:E66. doi:10.5888/pcd10.120239 23618546 PMC3652713

[59] Rocca WA, Boyd CM, Grossardt BR, . Prevalence of multimorbidity in a geographically defined American population: patterns by age, sex, and race/ethnicity. Mayo Clin Proc. 2014;89(10):1336-1349. doi:10.1016/j.mayocp.2014.07.010 25220409 PMC4186914

[60] St Sauver JL, Chamberlain AM, Bobo WV, . Implementing the US Department of Health and Human Services definition of multimorbidity: a comparison between billing codes and medical record review in a population-based sample of persons 40-84 years old. BMJ Open. 2021;11(4):e042870. doi:10.1136/bmjopen-2020-042870 33895712 PMC8074567

[61] Quinones AR, Botoseneanu A, Markwardt S, . Racial/ethnic differences in multimorbidity development and chronic disease accumulation for middle-aged adults. PLoS One. 2019;14(6):e0218462. doi:10.1371/journal.pone.0218462 31206556 PMC6576751

[62] Harder VS, Stuart EA, Anthony JC. Propensity score techniques and the assessment of measured covariate balance to test causal associations in psychological research. Psychol Methods. 2010;15(3):234-249. doi:10.1037/a0019623 20822250 PMC2936698

[63] Rubin DB. The design versus the analysis of observational studies for causal effects: parallels with the design of randomized trials. Stat Med. 2007;26(1):20-36. doi:10.1002/sim.2739 17072897

[64] Andersson T, Alfredsson L, Källberg H, Zdravkovic S, Ahlbom A. Calculating measures of biological interaction. Eur J Epidemiol. 2005;20(7):575-579. doi:10.1007/s10654-005-7835-x 16119429

[65] Li R, Chambless L. Test for additive interaction in proportional hazards models. Ann Epidemiol. 2007;17(3):227-236. doi:10.1016/j.annepidem.2006.10.009 17320789

[66] Wulsin LR, Singal BM. Do depressive symptoms increase the risk for the onset of coronary disease: a systematic quantitative review. Psychosom Med. 2003;65(2):201-210. doi:10.1097/01.PSY.0000058371.50240.E3 12651987

[67] Nicholson A, Kuper H, Hemingway H. Depression as an aetiologic and prognostic factor in coronary heart disease: a meta-analysis of 6362 events among 146 538 participants in 54 observational studies. Eur Heart J. 2006;27(23):2763-2774. doi:10.1093/eurheartj/ehl338 17082208

[68] Coryell W, Noyes R Jr, House JD. Mortality among outpatients with anxiety disorders. Am J Psychiatry. 1986;143(4):508-510. doi:10.1176/ajp.143.4.508 3953892

[69] Ferketich AK, Schwartzbaum JA, Frid DJ, Moeschberger ML. Depression as an antecedent to heart disease among women and men in the NHANES I study: National Health and Nutrition Examination Survey. Arch Intern Med. 2000;160(9):1261-1268. doi:10.1001/archinte.160.9.1261 10809028

[70] Farmer A, Korszun A, Owen MJ, . Medical disorders in people with recurrent depression. Br J Psychiatry. 2008;192(5):351-355. doi:10.1192/bjp.bp.107.038380 18450658

[71] Poole L, Steptoe A. Depressive symptoms predict incident chronic disease burden 10 years later: findings from the English Longitudinal Study of Ageing (ELSA). J Psychosom Res. 2018;113:30-36. doi:10.1016/j.jpsychores.2018.07.009 30190045

[72] Xu X, Mishra GD, Jones M. Depressive symptoms and the development and progression of physical multimorbidity in a national cohort of Australian women. Health Psychol. 2019;38(9):812-821. doi:10.1037/hea0000738 31144827

[73] Uijen AA, van de Lisdonk EH. Multimorbidity in primary care: prevalence and trend over the last 20 years. Eur J Gen Pract. 2008;14(suppl 1):28-32. doi:10.1080/13814780802436093 18949641

[74] Niles AN, O’Donovan A. Comparing anxiety and depression to obesity and smoking as predictors of major medical illnesses and somatic symptoms. Health Psychol. 2019;38(2):172-181. doi:10.1037/hea0000707 30556708 PMC6592048

[75] Kessler RC, Nelson CB, McGonagle KA, Liu J, Swartz M, Blazer DG. Comorbidity of *DSM-III-R* major depressive disorder in the general population: results from the US National Comorbidity Survey. Br J Psychiatry Suppl. 1996;168(30):17-30. doi:10.1192/S0007125000298371 8864145

[76] Wittchen H-U, Nelson CB, Lachner G. Prevalence of mental disorders and psychosocial impairments in adolescents and young adults. Psychol Med. 1998;28(1):109-126. doi:10.1017/S0033291797005928 9483687

[77] Fabbri E, Zoli M, Gonzalez-Freire M, Salive ME, Studenski SA, Ferrucci L. Aging and multimorbidity: new tasks, priorities, and frontiers for integrated gerontological and clinical research. J Am Med Dir Assoc. 2015;16(8):640-647. doi:10.1016/j.jamda.2015.03.013 25958334 PMC5125299

[78] Miller AH, Raison CL. The role of inflammation in depression: from evolutionary imperative to modern treatment target. Nat Rev Immunol. 2016;16(1):22-34. doi:10.1038/nri.2015.5 26711676 PMC5542678

[79] Leonard BE, Myint A. The psychoneuroimmunology of depression. Hum Psychopharmacol. 2009;24(3):165-175. 19212943 10.1002/hup.1011

[80] Maes M, Kubera M, Obuchowiczwa E, Goehler L, Brzeszcz J. Depression’s multiple comorbidities explained by (neuro)inflammatory and oxidative & nitrosative stress pathways. Neuro Endocrinol Lett. 2011;32(1):7-24. 21407167

[81] Gardner A, Boles RG. Beyond the serotonin hypothesis: mitochondria, inflammation and neurodegeneration in major depression and affective spectrum disorders. Prog Neuropsychopharmacol Biol Psychiatry. 2011;35(3):730-743. doi:10.1016/j.pnpbp.2010.07.030 20691744

[82] Triolo F, Harber-Aschan L, Belvederi Murri M, . The complex interplay between depression and multimorbidity in late life: risks and pathways. Mech Ageing Dev. 2020;192:111383. doi:10.1016/j.mad.2020.111383 33045250

[83] Schmidt HD, Shelton RC, Duman RS. Functional biomarkers of depression: diagnosis, treatment, and pathophysiology. Neuropsychopharmacology. 2011;36(12):2375-2394. doi:10.1038/npp.2011.151 21814182 PMC3194084

[84] St John PD, Tyas SL, Menec V, Tate R. Multimorbidity, disability, and mortality in community-dwelling older adults. Can Fam Physician. 2014;60(5):e272-e280. 24829022 PMC4020665

[85] Wolff JL, Boult C, Boyd C, Anderson G. Newly reported chronic conditions and onset of functional dependency. J Am Geriatr Soc. 2005;53(5):851-855. doi:10.1111/j.1532-5415.2005.53262.x 15877563

[86] Spijker J, Muntingh A, Batelaan N. Advice for clinicians on how to treat comorbid anxiety and depression. JAMA Psychiatry. 2020;77(6):645-646. doi:10.1001/jamapsychiatry.2020.0601 32293658

[87] Gaspersz R, Lamers F, Beekman ATF, van Hemert AM, Schoevers RA, Penninx BWJH. The impact of depressive disorder symptoms and subtypes on 6-Year incidence of somatic diseases. Psychother Psychosom. 2018;87(5):308-310. doi:10.1159/000491933 30114682 PMC6219696

[88] Altemus M, Sarvaiya N, Neill Epperson C. Sex differences in anxiety and depression clinical perspectives. Front Neuroendocrinol. 2014;35(3):320-330. doi:10.1016/j.yfrne.2014.05.004 24887405 PMC4890708

[89] Rosellini AJ, Bourgeois ML, Correa J, Tung ES, Goncharenko S, Brown TA. Anxious distress in depressed outpatients: prevalence, comorbidity, and incremental validity. J Psychiatr Res. 2018;103:54-60. doi:10.1016/j.jpsychires.2018.05.006 29778071 PMC8903047

[90] Agency for Healthcare Research and Quality; National Center for Health Statistics. Appendix A—clinical classification software-diagnoses (January 1980 through September 2013). Revised March 2016. Accessed February 22, 2022. https://www.hcup-us.ahrq.gov/toolssoftware/ccs/AppendixASingleDX.txt

[91] American Psychiatric Association. Diagnostic and Statistical Manual of Mental Disorders. 5th ed. American Psychiatric Association; 2013.

[92] Etkin A, Wager TD. Functional neuroimaging of anxiety: a meta-analysis of emotional processing in PTSD, social anxiety disorder, and specific phobia. Am J Psychiatry. 2007;164(10):1476-1488. doi:10.1176/appi.ajp.2007.07030504 17898336 PMC3318959

[93] Shalev AY, Bonne O, Eth S. Treatment of posttraumatic stress disorder: a review. Psychosom Med. 1996;58(2):165-182. doi:10.1097/00006842-199603000-00012 8849635

[94] Karakus MC, Patton LC. Depression and the onset of chronic illness in older adults: a 12-year prospective study. J Behav Health Serv Res. 2011;38(3):373-382. doi:10.1007/s11414-011-9234-2 21293976

[95] Violan C, Foguet-Boreu Q, Flores-Mateo G, . Prevalence, determinants and patterns of multimorbidity in primary care: a systematic review of observational studies. PLoS One. 2014;9(7):e102149. doi:10.1371/journal.pone.0102149 25048354 PMC4105594

[96] Mauvais-Jarvis F, Bairey Merz N, Barnes PJ, . Sex and gender: modifiers of health, disease, and medicine. Lancet. 2020;396(10250):565-582. doi:10.1016/S0140-6736(20)31561-0 32828189 PMC7440877

[97] Goldstein JM, Langer A, Lesser JA. Sex differences in disorders of the brain and heart—a global crisis of multimorbidity and novel opportunity. JAMA Psychiatry. 2021;78(1):7-8. doi:10.1001/jamapsychiatry.2020.1944 32639554 PMC8612029

[98] Langer A, Meleis A, Knaul FM, . Women and Health: the key for sustainable development. Lancet. 2015;386(9999):1165-1210. doi:10.1016/S0140-6736(15)60497-4 26051370

[99] Li SH, Graham BM. Why are women so vulnerable to anxiety, trauma-related and stress-related disorders: the potential role of sex hormones. Lancet Psychiatry. 2017;4(1):73-82. doi:10.1016/S2215-0366(16)30358-3 27856395

[100] Rainville JR, Hodes GE. Inflaming sex differences in mood disorders. Neuropsychopharmacology. 2019;44(1):184-199. doi:10.1038/s41386-018-0124-7 29955150 PMC6235877

[101] Renna ME, O’Toole MS, Spaeth PE, Lekander M, Mennin DS. The association between anxiety, traumatic stress, and obsessive-compulsive disorders and chronic inflammation: a systematic review and meta-analysis. Depress Anxiety. 2018;35(11):1081-1094. doi:10.1002/da.22790 30199144

[102] Wohleb ES, Franklin T, Iwata M, Duman RS. Integrating neuroimmune systems in the neurobiology of depression. Nat Rev Neurosci. 2016;17(8):497-511. doi:10.1038/nrn.2016.69 27277867

[103] Hotamisligil GS. Inflammation and metabolic disorders. Nature. 2006;444(7121):860-867. doi:10.1038/nature05485 17167474

[104] Liu Y-Z, Wang Y-X, Jiang C-L. Inflammation: the common pathway of stress-related diseases. Front Hum Neurosci. 2017;11:316. doi:10.3389/fnhum.2017.00316 28676747 PMC5476783

[105] Slavich GM, Irwin MR. From stress to inflammation and major depressive disorder: a social signal transduction theory of depression. Psychol Bull. 2014;140(3):774-815. doi:10.1037/a0035302 24417575 PMC4006295

[106] Bor W, Dean AJ, Najman J, Hayatbakhsh R. Are child and adolescent mental health problems increasing in the 21st century: a systematic review. Aust N Z J Psychiatry. 2014;48(7):606-616. doi:10.1177/0004867414533834 24829198

[107] Mojtabai R, Olfson M, Han B. National trends in the prevalence and treatment of depression in adolescents and young adults. Pediatrics. 2016;138(6):e20161878. doi:10.1542/peds.2016-1878 27940701 PMC5127071

[108] Call JB, Shafer K. Gendered manifestations of depression and help seeking among men. Am J Mens Health. 2018;12(1):41-51. doi:10.1177/1557988315623993 26721265 PMC5734537

[109] Goodman RA, Posner SF, Huang ES, Parekh AK, Koh HK. Defining and measuring chronic conditions: imperatives for research, policy, program, and practice. Prev Chronic Dis. 2013;10:E66. doi:10.5888/pcd10.120239 23618546 PMC3652713

[110] Diederichs C, Berger K, Bartels DB. The measurement of multiple chronic diseases—a systematic review on existing multimorbidity indices. J Gerontol A Biol Sci Med Sci. 2011;66(3):301-311. doi:10.1093/gerona/glq208 21112963

[111] Koyanagi A, Köhler-Forsberg O, Benros ME, . Mortality in unipolar depression preceding and following chronic somatic diseases. Acta Psychiatr Scand. 2018;138(6):500-508. doi:10.1111/acps.12899 29761489

[112] Dokucu ME, Cloninger CR. Personality disorders and physical comorbidities: a complex relationship. Curr Opin Psychiatry. 2019;32(5):435-441. doi:10.1097/YCO.0000000000000536 31219842

