# Supplementary material for: Association of Depression and Anxiety With the Accumulation of Chronic Conditions
Source: JAMA Netw Open. 2022 May 2;5(5):e229817. doi: 10.1001/jamanetworkopen.2022.9817 (PMC9062691; doi:10.1001/jamanetworkopen.2022.9817)
Supplement: Supplement. — eTable 1. List of the Diagnosis Codes Used to Define Depression and Anxiety eTable 2. List of Chronic Conditions and Corresponding Diagnosis Codes eTable 3. Risk of Death in Birthday Age Cohorts for Women and Men Separately eFigure 1. Inclusion of Individuals in Birthday Age Cohorts and Accumulation of Chronic Conditions eFigure 2. Balance of Characteristics by Birthday Age Cohort eFigure 3. Kaplan-Meier Estimates of Mortality in the Age 60 Years Cohort Separately for Women and Men [file jamanetwopen-e229817-s001.pdf]

Supplemental Online Content

Bobo WV, Grossardt BR, Virani S, St Sauver JL, Boyd CM, Rocca WA. Association of depression and anxiety with the accumulation of chronic conditions. *JAMA Netw Open*. 2022;5(5):e229817. doi:10.1001/jamanetworkopen.2022.9817

eTable 1. List of the Diagnosis Codes Used to Define Depression and Anxiety

eTable 2. List of Chronic Conditions and Corresponding Diagnosis Codes

eTable 3. Risk of Death in Birthday Age Cohorts for Women and Men Separately

eFigure 1. Inclusion of Individuals in Birthday Age Cohorts and Accumulation of Chronic Conditions

eFigure 2. Balance of Characteristics by Birthday Age Cohort

eFigure 3. Kaplan-Meier Estimates of Mortality in the Age 60 Years Cohort Separately for Women and Men

This supplemental material has been provided by the authors to give readers additional information about their work.

eTable 1. List of the Diagnosis Codes Used to Define Depression and Anxiety

Condition description	CCS category	CMS (ICD-9 Codes)
Depression [a]	657	296.20, 296.21, 296.22, 296.23, 296.24, 296.25, 296.26, 296.30, 296.31, 296.32, 296.33, 296.34, 296.35, 296.36, 296.51, 296.52, 296.53, 296.54, 296.55, 296.56, 296.60, 296.61, 296.62, 296.63, 296.64, 296.65, 296.66, 296.89, 298.0, 300.4, 309.1, 311
Anxiety [b]	651	Not applicable

CCS =Clinical Classifications Software; CMS = Centers for Medicare and Medicaid Services; ICD = International Classification of Diseases.

[a] Depressive disorders were defined by the US Department of Health and Human Services as detailed elsewhere (http://www.cdc.gov/pcd/issues/2013/12_0239.htm). Depression is defined by having two codes separated by more than 30 days from among either the Clinical Classifications Software group of codes (developed by the Agency for Healthcare Research and Quality) or the Centers for Medicare and Medicaid Services group of codes (https://www.ccwdata.org/web/guest/condition-categories).

[b] Anxiety disorders were not included among the 20 conditions identified by the US Department of Health and Human Services; however, they are an additional important group of conditions affecting persons at most ages, and have been included in previous publication from our group.[16] Anxiety is defined by having two codes separated by more than 30 days from among the ICD-9 codes that make up the Clinical Classifications Software category number 651.

© 2022 Bobo WV et al. *JAMA Network Open.*

eTable 2. List of Chronic Conditions and Corresponding Diagnosis Codes

Condition description	CCS	CMS (ICD-9 Codes)	CMS (ICD-10 Codes; *Nov 2017 version*)
Hypertension	98, 99	401.0, 401.1, 401.9, 402.00, 402.01, 402.10, 402.11, 402.90, 402.91, 403.00, 403.01, 403.10, 403.11, 403.90, 403.91, 404.00, 404.01, 404.02, 404.03, 404.10, 404.11, 404.12, 404.13, 404.90, 404.91, 404.92, 404.93, 405.01, 405.09, 405.11, 405.19, 405.91, 405.99, 362.11, 437.2	H35.031, H35.032, H35.033, H35.039, I10, I11.0, I11.9, I12.0, I12.9, I13.0, I13.10, I13.11, I13.2, I15.0, I15.1, I15.2, I15.8, I15.9, I67.4, N26.2
Congestive heart failure	108	398.91, 402.01, 402.11, 402.91, 404.01, 404.11, 404.91, 404.03, 404.13, 404.93, 428.0, 428.1, 428.20, 428.21, 428.22, 428.23, 428.30, 428.31, 428.32, 428.33, 428.40, 428.41, 428.42, 428.43, 428.9	I09.81, I11.0, I13.0, I13.2, I50.1, I50.20, I50.21, I50.22, I50.23, I50.30, I50.31, I50.32, I50.33, I50.40, I50.41, I50.42, I50.43, I50.810, I50.811, I50.812, I50.813, I50.814, I50.82, I50.83, I50.84, I50.89, I50.9
Coronary artery disease	100, 101	410.00, 410.01, 410.02, 410.10, 410.11, 410.12, 410.20, 410.21, 410.22, 410.30, 410.31, 410.32, 410.40, 410.41, 410.42, 410.50, 410.51, 410.52, 410.60, 410.61, 410.62, 410.70, 410.71, 410.72, 410.80, 410.81, 410.82, 410.90, 410.91, 410.92, 411.0, 411.1, 411.81, 411.89, 412, 413.0, 413.1, 413.9, 414.00, 414.01, 414.02, 414.03, 414.04, 414.05, 414.06, 414.07, 414.12, 414.2, 414.3, 414.8, 414.9	I20.0, I20.1, I20.8, I20.9, I21.01, I21.02, I21.09, I21.11, I21.19, I21.21, I21.29, I21.3, I21.4, I21.A1, I21.A9, I22.0, I22.1, I22.2, I22.8, I22.9, I23.0, I23.1, I23.2, I23.3, I23.4, I23.5, I23.6, I23.7, I23.8, I24.0, I24.1, I24.8, I24.9, I25.10, I25.110, I25.111, I25.118, I25.119, I25.2, I25.3, I25.41, I25.42, I25.5, I25.6, I25.700, I25.701, I25.708, I25.709, I25.710, I25.711, I25.718, I25.719, I25.720, I25.721, I25.728, I25.729, I25.730, I25.731, I25.738, I25.739, I25.750, I25.751, I25.758, I25.759, I25.760, I25.761, I25.768, I25.769, I25.790, I25.791, I25.798, I25.799, I25.810, I25.811, I25.812, I25.82, I25.83, I25.84, I25.89, I25.9
Cardiac arrhythmias	105, 106	427.31	I48.0, I48.1, I48.2, I48.91
Hyperlipidemia	53	272.0, 272.1, 272.2, 272.3, 272.4	E78.0, E78.1, E78.2, E78.3, E78.4, E78.5
Stroke (including TIAs)	109–112	430, 431, 433.01, 433.11, 433.21, 433.31, 433.81, 433.91, 434.00, 434.01,434.10, 434.11, 434.90, 434.91, 435.0, 435.1, 435.3, 435.8, 435.9, 436, 997.02	G45.0, G45.1, G45.2, G45.8, G45.9, G46.0, G46.1, G46.2, G46.3, G46.4, G46.5, G46.6, G46.7, G46.8, G97.31, G97.32, I60.00, I60.01, I60.02, I60.10, I60.11, I60.12, I60.20, I60.21, I60.22, I60.30, I60.31, I60.32, I60.4, I60.50, I60.51, I60.52, I60.6, I60.7, I60.8, I60.9, I61.0, I61.1, I61.2, I61.3, I61.4, I61.5, I61.6, I61.8, I61.9, I63.00, I63.02, I63.011, I63.012, I63.013, I63.019, I63.02, I63.031, I63.032, I63.039, I63.09, I63.10, I63.111, I63.112, I63.119, I63.12, I63.131, I63.132, I63.139, I63.19, I63.20, I63.211, I63.212, I63.213, I63.219, I63.22, I63.231, I63.232, I63.233, I63.239, I63.29, I63.30, I63.311, I63.312, I63.313, I63.319, I63.321, I63.322, I63.323, I63.329, I63.331, I63.332, I63.333, I63.339, I63.341, I63.342, I63.343, I63.349, I63.39, I63.40, I63.411, I63.412, I63.413, I63.419, I63.421, I63.422, I63.423, I63.429, I63.431, I63.432, I63.433, I63.439, I63.441, I63.442, I63.443, I63.449, I63.49, I63.50, I63.511, I63.512, I63.513, I63.519, I63.521, I63.522, I63.523, I63.529, I63.531, I63.532, I63.533, I63.539, I63.541, I63.542, I63.543, I63.549, I63.59, I63.6, I63.8, I63.9, I66.01, I66.02, I66.03, I66.09, I66.11, I66.12, I66.13, I66.19, I66.21, I66.22, I66.23, I66.29, I66.3, I66.8, I66.9, I67.841, I67.848, I67.89, I97.810, I97.811, I97.820, I97.821
Arthritis *(continued on next page)*	202, 203	714.0, 714.1, 714.2, 714.30, 714.31, 714.32, 714.33, 715.00, 715.04, 715.09, 715.10, 715.11, 715.12, 715.13, 715.14, 715.15, 715.16, 715.17, 715.18, 715.20, 715.21, 715.22, 715.23, 715.24, 715.25, 715.26, 715.27, 715.28, 715.30, 715.31, 715.32, 715.33, 715.34, 715.35, 715.36, 715.37, 715.38, 715.80, 715.89, 715.90, 715.91, 715.92, 715.93, 715.94, 715.95, 715.96, 715.97, 715.98, 720.0, 721.0, 721.1, 721.2, 721.3, 721.90, 721.91	M05.00, M05.011, M05.012, M05.019, M05.021, M05.022, M05.029, M05.031, M05.032, M05.039, M05.041, M05.042, M05.049, M05.051, M05.052, M05.059, M05.061, M05.062, M05.069, M05.071, M05.072, M05.079, M05.09, M05.20, M05.211, M05.212, M05.219, M05.221, M05.222, M05.229, M05.231, M05.232, M05.239, M05.241, M05.242, M05.249, M05.251, M05.252, M05.259, M05.261, M05.262, M05.269, M05.271, M05.272, M05.279, M05.29, M05.30, M05.311, M05.312, M05.319, M05.321, M05.322, M05.329, M05.331, M05.332, M05.339, M05.341, M05.342, M05.349, M05.351, M05.352, M05.359, M05.361, M05.362, M05.369, M05.371, M05.372, M05.379, M05.39, M05.40, M05.411, M05.412, M05.419, M05.421, M05.422, M05.429, M05.431, M05.432, M05.439, M05.441, M05.442, M05.449, M05.451, M05.452, M05.459, M05.461, M05.462, M05.469, M05.471, M05.472, M05.479, M05.49, M05.50, M05.511, M05.512, M05.519, M05.521, M05.522, M05.529, M05.531, M05.532, M05.539, M05.541, M05.542, M05.549, M05.551, M05.552, M05.559, M05.561, M05.562, M05.569, M05.571, M05.572, M05.579, M05.59, M05.60, M05.611, M05.612, M05.619, M05.621, M05.622, M05.629, M05.631, M05.632, M05.639, M05.641, M05.642, M05.649, M05.651, M05.652, M05.659, M05.661, M05.662, M05.669, M05.671, M05.672, M05.679, M05.69, M05.70, M05.711, M05.712, M05.719, M05.721, M05.722, M05.729, M05.731, M05.732, M05.739, M05.741, M05.742, M05.749, M05.751, M05.752, M05.759, M05.761, M05.762, M05.769, M05.771, M05.772, M05.779, M05.79, M05.80, M05.811, M05.812, M05.819, M05.821, M05.822, M05.829, M05.831, M05.832, M05.839, M05.841, M05.842, M05.849, M05.851, M05.852, M05.859, M05.861, M05.862, M05.869, M05.871, M05.872, M05.879, M05.89, M05.9, M06.00, M06.011, M06.012, M06.019, M06.021, M06.022, M06.029, M06.031, M06.032, M06.039, M06.041, M06.042, M06.049, M06.051, M06.052, M06.059, M06.061, M06.062, M06.069, M06.071, M06.072, M06.079, M06.08, M06.09, M06.1, M06.20, M06.211, M06.212, M06.219, M06.221, M06.222, M06.229, M06.231, M06.232, M06.239, M06.241, M06.242, M06.249, M06.251, M06.252, M06.259, M06.261, M06.262, M06.269, M06.271, M06.272, M06.279, M06.28, M06.29, M06.30, M06.311, M06.312, M06.319, M06.321, M06.322, M06.329, M06.331, M06.332, M06.339, M06.341, M06.342, M06.349, M06.351, M06.352, M06.359, M06.361, M06.362, M06.369, M06.371, M06.372, M06.379, M06.38, M06.39, M06.80, M06.811, M06.812, M06.819, M06.821, M06.822, M06.829, M06.831, M06.832, M06.839, M06.841, M06.842, M06.849, M06.851, M06.852, M06.859, M06.861, M06.862, M06.869, M06.871, M06.872, M06.879, M06.88, M06.89, M06.9, M08.00, M08.011, M08.012, M08.019, M08.021, M08.022, M08.029, M08.031, M08.032, M08.039, M08.041,

© 2022 Bobo WV et al. *JAMA Network Open.*

eTable 2. *(continued)*

Condition description	CCS	CMS (ICD-9 Codes)	CMS (ICD-10 Codes; *Nov 2017 version*)
Arthritis *(continued)*	---	---	M08.042, M08.049, M08.051, M08.052, M08.059, M08.061, M08.062, M08.069, M08.071, M08.072, M08.079, M08.08, M08.09, M08.1, M08.20, M08.211, M08.212, M08.219, M08.221, M08.222, M08.229, M08.231, M08.232, M08.239, M08.241, M08.242, M08.249, M08.251, M08.252, M08.259, M08.261, M08.262, M08.269, M08.271, M08.272, M08.279, M08.28, M08.29, M08.3, M08.40, M08.411, M08.412, M08.419, M08.421, M08.422, M08.429, M08.431, M08.432, M08.439, M08.441, M08.442, M08.449, M08.451, M08.452, M08.459, M08.461, M08.462, M08.469, M08.471, M08.472, M08.479, M08.48, M08.80, M08.811, M08.812, M08.819, M08.821, M08.822, M08.829, M08.831, M08.832, M08.839, M08.841, M08.842, M08.849, M08.851, M08.852, M08.859, M08.861, M08.862, M08.869, M08.871, M08.872, M08.879, M08.88, M08.89, M08.90, M08.911, M08.912, M08.919, M08.921, M08.922, M08.929, M08.931, M08.932, M08.939, M08.941, M08.942, M08.949, M08.951, M08.952, M08.959, M08.961, M08.962, M08.969, M08.971, M08.972, M08.979, M08.98, M08.99, M15.0, M15.1, M15.2, M15.3, M15.4, M15.8, M15.9, M16.0, M16.10, M16.11, M16.12, M16.2, M16.30, M16.31, M16.32, M16.4, M16.50, M16.51, M16.52, M16.6, M16.7, M16.9, M17.0, M17.10, M17.11, M17.12, M17.2, M17.30, M17.31, M17.32, M17.4, M17.5, M17.9, M18.0, M18.10, M18.11, M18.12, M18.2, M18.30, M18.31, M18.32, M18.4, M18.50, M18.51, M18.52, M18.9, M19.011, M19.012, M19.019, M19.021, M19.022, M19.029, M19.031, M19.032, M19.039, M19.041, M19.042, M19.049, M19.071, M19.072, M19.079, M19.111, M19.112, M19.119, M19.121, M19.122, M19.129, M19.131, M19.132, M19.139, M19.141, M19.142, M19.149, M19.171, M19.172, M19.179, M19.211, M19.212, M19.219, M19.221, M19.222, M19.229, M19.231, M19.232, M19.239, M19.241, M19.242, M19.249, M19.271, M19.272, M19.279, M19.90, M19.91, M19.92, M19.93, M45.0, M45.1, M45.2, M45.3, M45.4, M45.5, M45.6, M45.7, M45.8, M45.9, M47.011, M47.012, M47.013, M47.014, M47.015, M47.016, M47.019, M47.021, M47.022, M47.029, M47.10, M47.11, M47.12, M47.13, M47.20, M47.21, M47.22, M47.23, M47.24, M47.25, M47.26, M47.27, M47.28, M47.811, M47.812, M47.813, M47.814, M47.815, M47.816, M47.817, M47.818, M47.819, M47.891, M47.892, M47.893, M47.894, M47.895, M47.896, M47.897, M47.898, M47.899, M47.9, M48.8X1, M48.8X2, M48.8X3, M48.8X4, M48.8X5, M48.8X6, M48.8X7, M48.8X8, M48.8X9
Asthma	128	493.00, 493.01, 493.02, 493.10, 493.11, 493.12, 493.20, 493.21, 493.22, 493.81, 493.82, 493.90, 493.91, 493.92	J45.20, J45.21, J45.22, J45.30, J45.31, J45.32, J45.40, J45.41, J45.42, J45.50, J45.51, J45.52, J45.901, J45.902, J45.909, J45.990, J45.991, J45.998
Cancer	11–43	**Breast cancer**: 174.0, 174.1, 174.2, 174.3, 174.4, 174.5, 174.6, 174.8, 174.9, 175.0, 175.9, 233.0, V10.3. **Colorectal cancer**: 154.0, 154.1, 153.0, 153.1, 153.2, 153.3, 153.4, 153.5, 153.6, 153.7, 153.8, 153.9, 230.3, 230.4, V10.05. **Prostate cancer**: 185, 233.4, V10.46. **Lung cancer**: 162.2, 162.3, 162.4, 162.5, 162.8, 162.9, 231.2, V10.11.	**Breast cancer**: C50.011, C50.012, C50.019, C50.021, C50.022, C50.029, C50.111, C50.112, C50.119, C50.121, C50.122, C50.129, C50.211, C50.212, C50.219, C50.221, C50.222, C50.229, C50.311, C50.312, C50.319, C50.321, C50.322, C50.329, C50.411, C50.412, C50.419, C50.421, C50.422, C50.429, C50.511, C50.512, C50.519, C50.521, C50.522, C50.529, C50.611, C50.612, C50.619, C50.621, C50.622, C50.629, C50.811, C50.812, C50.819, C50.821, C50.822, C50.829, C50.911, C50.912, C50.919, C50.921, C50.922, C50.929, D05.00, D05.01, D05.02, D05.10, D05.11, D05.12, D05.80, D05.81, D05.82, D05.90, D05.91, D05.92, Z85.3 **Colorectal cancer**: C18.0, C18.1, C18.2, C18.3, C18.4, C18.5, C18.6, C18.7, C18.8, C18.9, C19, C20, D01.0, D01.1, D01.2, Z85.038, Z85.040, Z85.048 **Prostate cancer**: C61, D07.5, Z85.46 **Lung cancer**: C34.00, C34.01, C34.02, C34.10, C34.11, C34.12, C34.2, C34.30, C34.31, C34.32, C34.80, C34.81, C34.82, C34.90, C34.91, C34.92, D02.20, D02.21, D02.22, Z85.110, Z85.118
Chronic kidney disease	158	016.00, 016.01, 016.02, 016.03, 016.04, 016.05, 016.06, 095.4, 189.0, 189.9, 223.0, 236.91, 249.40, 249.41, 250.40, 250.41, 250.42, 250.43, 271.4, 274.10, 283.11, 403.01, 403.11, 403.91, 404.02, 404.03, 404.12, 404.13, 404.92, 404.93, 440.1, 442.1, 572.4, 580.0, 580.4, 580.81, 580.89, 580.9, 581.0, 581.1, 581.2, 581.3, 581.81, 581.89, 581.9, 582.0, 582.1, 582.2, 582.4, 582.81, 582.89, 582.9, 583.0, 583.1, 583.2, 583.4, 583.6, 583.7, 583.81, 583.89, 583.9, 584.5, 584.6, 584.7, 584.8, 584.9, 585.1, 585.2, 585.3, 585.4, 585.5, 585.6, 585.9, 586, 587, 588.0, 588.1, 588.81, 588.89, 588.9, 591, 753.12, 753.13, 753.14, 753.15, 753.16, 753.17, 753.19, 753.20, 753.21, 753.22, 753.23, 753.29, 794.4	A18.11, A52.75, B52.0, C64.1, C64.2, C64.9, C68.9, D30.00, D30.01, D30.02, D41.00, D41.01, D41.02, D41.10, D41.11, D41.12, D41.20, D41.21, D41.22, D59.3, E08.21, E08.22, E08.29, E08.65, E09.21, E09.22, E09.29, E10.21, E10.22, E10.29, E10.65, E11.21, E11.22, E11.29, E11.65, E13.21, E13.22, E13.29, E74.8, I12.0, I12.9, I13.0, I13.10, I13.11, I13.2, I70.1, I72.2, K76.7, M10.30, M10.311, M10.312, M10.319, M10.321, M10.322, M10.329, M10.331, M10.332, M10.339, M10.341, M10.342, M10.349, M10.351, M10.352, M10.359, M10.361, M10.362, M10.369, M10.371, M10.372, M10.379, M10.38, M10.39, M32.14, M32.15, M35.04, N00.0, N00.1, N00.2, N00.3, N00.4, N00.5, N00.6, N00.7, N00.8, N00.9, N01.0, N01.1, N01.2, N01.3, N01.4, N01.5, N01.6, N01.7, N01.8, N01.9, N02.0, N02.1, N02.2, N02.3, N02.4, N02.5, N02.6, N02.7, N02.8, N02.9, N03.0, N03.1, N03.2, N03.3, N03.4, N03.5, N03.6, N03.7, N03.8, N03.9, N04.0, N04.1, N04.2, N04.3, N04.4, N04.5, N04.6, N04.7, N04.8, N04.9, N05.0, N05.1, N05.2, N05.3, N05.4, N05.5, N05.6, N05.7, N05.8, N05.9, N06.0, N06.1, N06.2, N06.3, N06.4, N06.5, N06.6, N06.7, N06.8, N06.9, N07.0, N07.1, N07.2, N07.3, N07.4, N07.5, N07.6, N07.7, N07.8, N07.9, N08, N13.1, N13.2, N13.30, N13.39, N14.0, N14.1, N14.2, N14.3, N14.4, N15.0, N15.8, N15.9, N16, N17.0, N17.1, N17.2, N17.8, N17.9, N18.1, N18.2, N18.3, N18.4, N18.5, N18.6, N18.9, N19, N25.0, N25.1, N25.81, N25.89, N25.9, N26.1, N26.9, Q61.02, Q61.11, Q61.19, Q61.2, Q61.3, Q61.4, Q61.5, Q61.8, Q62.0, Q62.2, Q62.10, Q62.11, Q62.12, Q62.31, Q62.32, Q62.39, R94.4
Chronic obstructive pulmonary disease	127	490, 491.0, 491.1, 491.20, 491.21, 491.22, 491.8, 491.9, 492.0, 492.8, 494.0, 494.1, 496	J40, J41.0, J41.1, J41.8, J42, J43.0, J43.1, J43.2, J43.8, J43.9, J44.0, J44.1, J44.9, J47.0, J47.1, J47.9

eTable 2. *(continued)*

Condition description	CCS	CMS (ICD-9 Codes)	CMS (ICD-10 Codes; *Nov 2017 version*)
Diabetes	49, 50	249.00, 249.01, 249.10, 249.11, 249.20, 249.21, 249.30, 249.31, 249.40, 249.41, 249.50, 249.51, 249.60, 249.61, 249.70, 249.71, 249.80, 249.81, 249.90, 249.91, 250.00, 250.01, 250.02, 250.03, 250.10, 250.11, 250.12, 250.13, 250.20, 250.21, 250.22, 250.23, 250.30, 250.31, 250.32, 250.33, 250.40, 250.41, 250.42, 250.43, 250.50, 250.51, 250.52, 250.53, 250.60, 250.61, 250.62, 250.63, 250.70, 250.71, 250.72, 250.73, 250.80, 250.81, 250.82, 250.83, 250.90, 250.91, 250.92, 250.93, 357.2, 362.01, 362.02, 362.03, 362.04, 362.05, 362.06, 366.41	E08.00, E08.01, E08.10, E08.11, E08.21, E08.22, E08.29, E08.311, E08.319, E08.321, E08.3211, E08.3212, E08.3213, E08.3219, E08.329, E08.3291, E08.3292, E08.3293, E08.3299, E08.331, E08.3311, E08.3312, E08.3313, E08.3319, E08.339, E08.3391, E08.3392, E08.3393, E08.3399, E08.341, E08.3411, E08.3412, E08.3413, E08.3419, E08.349, E08.3491, E08.3492, E08.3493, E08.3499, E08.351, E08.3511, E08.3512, E08.3513, E08.3519, E08.3521, E08.3522, E08.3523, E08.3529, E08.3531, E08.3532, E08.3533, E08.3539, E08.3541, E08.3542, E08.3543, E08.3549, E08.3551, E08.3552, E08.3553, E08.3559, E08.359, E08.3591, E08.3592, E08.3593, E08.3599, E08.36, E08.37X1, E08.37X2, E08.37X3, E08.37X9, E08.39, E08.40, E08.41, E08.42, E08.43, E08.44, E08.49, E08.51, E08.52, E08.59, E08.610, E08.618, E08.620, E08.621, E08.622, E08.628, E08.630, E08.638, E08.641, E08.649, E08.65, E08.69, E08.8, E08.9, E09.00, E09.01, E09.10, E09.11, E09.21, E09.22, E09.29, E09.311, E09.319, E09.321, E09.3211, E09.3212, E09.3213, E09.3219, E09.329, E09.3291, E09.3292, E09.3293, E09.3299, E09.331, E09.3311, E09.3312, E09.3313, E09.3319, E09.339, E09.3391, E09.3392, E09.3393, E09.3399, E09.341, E09.3411, E09.3412, E09.3413, E09.3419, E09.349, E09.3491, E09.3492, E09.3493, E09.3499, E09.351, E09.3511, E09.3512, E09.3513, E09.3519, E09.3521, E09.3522, E09.3523, E09.3529, E09.3531, E09.3532, E09.3533, E09.3539, E09.3541, E09.3542, E09.3543, E09.3549, E09.3551, E09.3552, E09.3553, E09.3559, E09.359, E09.3591, E09.3592, E09.3593, E09.3599, E09.36, E09.37X1, E09.37X2, E09.37X3, E09.37X9, E09.39, E09.40, E09.41, E09.42, E09.43, E09.44, E09.49, E09.51, E09.52, E09.59, E09.610, E09.618, E09.620, E09.621, E09.622, E09.628, E09.630, E09.638, E09.641, E09.649, E09.65, E09.69, E09.8, E09.9, E10.10, E10.11, E10.21, E10.22, E10.29, E10.311, E10.319, E10.321, E10.3211, E10.3212, E10.3213, E10.3219, E10.329, E10.3291, E10.3292, E10.3293, E10.3299, E10.331, E10.3311, E10.3312, E10.3313, E10.3319, E10.339, E10.3391, E10.3392, E10.3393, E10.3399, E10.341, E10.3411, E10.3412, E10.3413, E10.3419, E10.349, E10.3491, E10.3492, E10.3493, E10.3499, E10.351, E10.3511, E10.3512, E10.3513, E10.3519, E10.359, E10.36, E10.37X1, E10.37X2, E10.37X3, E10.37X9, E10.39, E10.40, E10.41, E10.42, E10.43, E10.44, E10.49, E10.51, E10.52, E10.59, E10.610, E10.618, E10.620, E10.621, E10.622, E10.628, E10.630, E10.638, E10.641, E10.649, E10.65, E10.69, E10.8, E10.9, E11.00, E11.01, E11.10, E11.11, E11.21, E11.22, E11.29, E11.311, E11.319, E11.321, E11.3211, E11.3212, E11.3213, E11.3219, E11.329, E11.3291, E11.3292, E11.3293, E11.3299, E11.331, E11.3311, E11.3312, E11.3313, E11.3319, E11.339, E11.3391, E11.3392, E11.3393, E11.3399, E11.341, E11.3411, E11.3412, E11.3413, E11.3419, E11.349, E11.3491, E11.3492, E11.3493, E11.3499, E11.351, E11.3511, E11.3512, E11.3513, E11.3519, E11.3521, E11.3522, E11.3523, E11.3529, E11.3531, E11.3532, E11.3533, E11.3539, E11.3541, E11.3542, E11.3543, E11.3549, E11.3551, E11.3552, E11.3553, E11.3559, E11.359, E11.3591, E11.3592, E11.3593, E11.3599, E11.36, E11.37X1, E11.37X2, E11.37X3, E11.37X9, E11.39, E11.40, E11.41, E11.42, E11.43, E11.44, E11.49, E11.51, E11.52, E11.59, E11.610, E11.618, E11.620, E11.621, E11.622, E11.628, E11.630, E11.638, E11.641, E11.649, E11.65, E11.69, E11.8, E11.9, E13.00, E13.01, E13.10, E13.11, E13.21, E13.22, E13.29, E13.311, E13.319, E13.321, E13.3211, E13.3212, E13.3213, E13.3219, E13.329, E13.3291, E13.3292, E13.3293, E13.3299, E13.331, E13.3311, E13.3312, E13.3313, E13.3319, E13.339, E13.3391, E13.3392, E13.3393, E13.3399, E13.341, E13.3411, E13.3412, E13.3413, E13.3419, E13.349, E13.3491, E13.3492, E13.3493, E13.3499, E13.351, E13.3511, E13.3512, E13.3513, E13.3519, E13.3521, E13.3522, E13.3523, E13.3529, E13.3531, E13.3532, E13.3533, E13.3539, E13.3541, E13.3542, E13.3543, E13.3549, E13.3551, E13.3552, E13.3553, E13.3559, E13.359, E13.36, E13.39, E13.40, E13.41, E13.42, E13.43, E13.44, E13.49, E13.51, E13.52, E13.59, E13.610, E13.618, E13.620, E13.621, E13.622, E13.628, E13.630, E13.638, E13.641, E13.649, E13.65, E13.69, E13.8, E13.9
Hepatitis	6	Not applicable	Not applicable
Human immunodeficiency virus (HIV)	5	Not applicable	Not applicable
Osteoporosis	206	733.00, 733.01, 733.02, 733.03, 733.09	M81.0, M81.6, M81.8

CCS =Clinical Classifications Software; CMS = Centers for Medicare and Medicaid Services; ICD = International Classification of Diseases.

The 15 chronic conditions were defined by the U.S. Department of Health and Human Services as detailed elsewhere (http://www.cdc.gov/pcd/issues/2013/12_0239.htm). Each condition is defined by having two codes separated by more than 30 days from among either the Clinical Classifications Software category of codes (developed by the Agency for Healthcare Research and Quality) or from the Centers for Medicare and Medicaid Services group of codes. Each CCS category includes a list of ICD-9 and ICD-10 codes as detailed elsewhere (https://www.ccwdata.org/web/guest/condition-categories). Some of the CCS lists of codes are extensive and not reproduced here (e.g., for cancers).

© 2022 Bobo WV et al. *JAMA Network Open.*

eTable 3. Risk of Death in Birthday Age Cohorts for Women and Men Separately

				Unadjusted analyses		Adjusted analyses [b]	
Cohort group	Persons	Persons-years	Deaths	HR (95% CI) [a]	p-value	HR (95% CI) [a]	p-value
Women							
Age 20 years cohort							
Referent	6,211	40,473	17	1 (ref)	---	1 (ref)	---
Depression alone	856	5,838	0	---[c]	---[c]	---[c]	---[c]
Anxiety alone	260	1,589	1	1.57 (0.21-11.78)	0.66	1.62 (0.26-10.15)	0.61
Both Dep. and Anx.	371	2,391	2	2.03 (0.47-8.79)	0.34	0.69 (0.07-6.51)	0.75
Age 40 years cohort							
Referent	5,356	40,948	21	1 (ref)	---	1 (ref)	---
Depression alone	1,036	7,614	11	2.83 (1.37-5.87)	0.005	2.35 (1.08-5.09)	0.03
Anxiety alone	361	2,578	1	0.76 (0.10-5.68)	0.79	0.09 (0.00-23.62)	0.39
Both Dep. and Anx.	377	2,520	7	5.47 (2.32-12.87)	0.0001	2.18 (0.67-7.13)	0.20
Age 60 years cohort							
Referent	5,279	38,549	162	1 (ref)	---	1 (ref)	---
Depression alone	901	6,309	43	1.66 (1.19-2.32)	0.003	1.42 (1.01-2.02)	0.047
Anxiety alone	221	1,592	5	0.77 (0.32-1.88)	0.57	0.77 (0.31-1.87)	0.56
Both Dep. and Anx.	287	1,940	17	2.16 (1.31-3.56)	0.003	1.24 (0.67-2.28)	0.50
Men							
Age 20 years cohort							
Referent	6,267	38,085	36	1 (ref)	---	1 (ref)	---
Depression alone	490	3,187	3	0.97 (0.30-3.16)	0.96	0.97 (0.30-3.19)	0.97
Anxiety alone	214	1,214	2	1.80 (0.43-7.50)	0.42	2.36 (0.69-8.10)	0.17
Both Dep. and Anx.	141	800	0	---[c]	---[c]	---[c]	---[c]
Age 40 years cohort							
Referent	5,139	37,776	54	1 (ref)	---	1 (ref)	---
Depression alone	407	2,822	6	1.56 (0.67-3.62)	0.31	1.69 (0.77-3.72)	0.19
Anxiety alone	239	1,617	4	1.84 (0.67-5.09)	0.24	0.98 (0.27-3.48)	0.97
Both Dep. and Anx.	145	935	3	2.42 (0.76-7.73)	0.14	1.74 (0.50-6.04)	0.38
Age 60 years cohort							
Referent	5,150	36,458	261	1 (ref)	---	1 (ref)	---
Depression alone	427	2,953	37	1.75 (1.24-2.47)	0.002	1.49 (1.04-2.15)	0.03
Anxiety alone	113	804	7	1.22 (0.58-2.58)	0.60	1.11 (0.52-2.40)	0.79
Both Dep. and Anx.	112	762	10	1.84 (0.98-3.46)	0.06	1.41 (0.68-2.93)	0.36

Anx. = Anxiety disorder; CI = confidence interval; Dep. = Depressive disorder; HR = hazards ratio.

[a] Time-to-event hazards ratios (HR) are estimated from Cox proportional hazards models.

[b] Adjusted mortality analyses are weighted using weights developed from generalized boosting models (GBM) to balance the four exposure groups at index birthday on potential confounders including calendar year of birthday, race, ethnicity, education, smoking status, BMI, and prevalence of each of the 15 chronic conditions (hypertension, congestive heart failure, coronary artery disease, cardiac arrhythmias, hyperlipidemia, stroke, arthritis, asthma, cancer, chronic kidney disease, chronic obstructive pulmonary disease, diabetes, hepatitis, HIV infection, and osteoporosis).

[c] HR was non-estimable because zero deaths were observed in the stratum.

© 2022 Bobo WV et al. *JAMA Network Open.*

eFigure 1. Inclusion of Individuals in Birthday Age Cohorts and Accumulation of Chronic Conditions

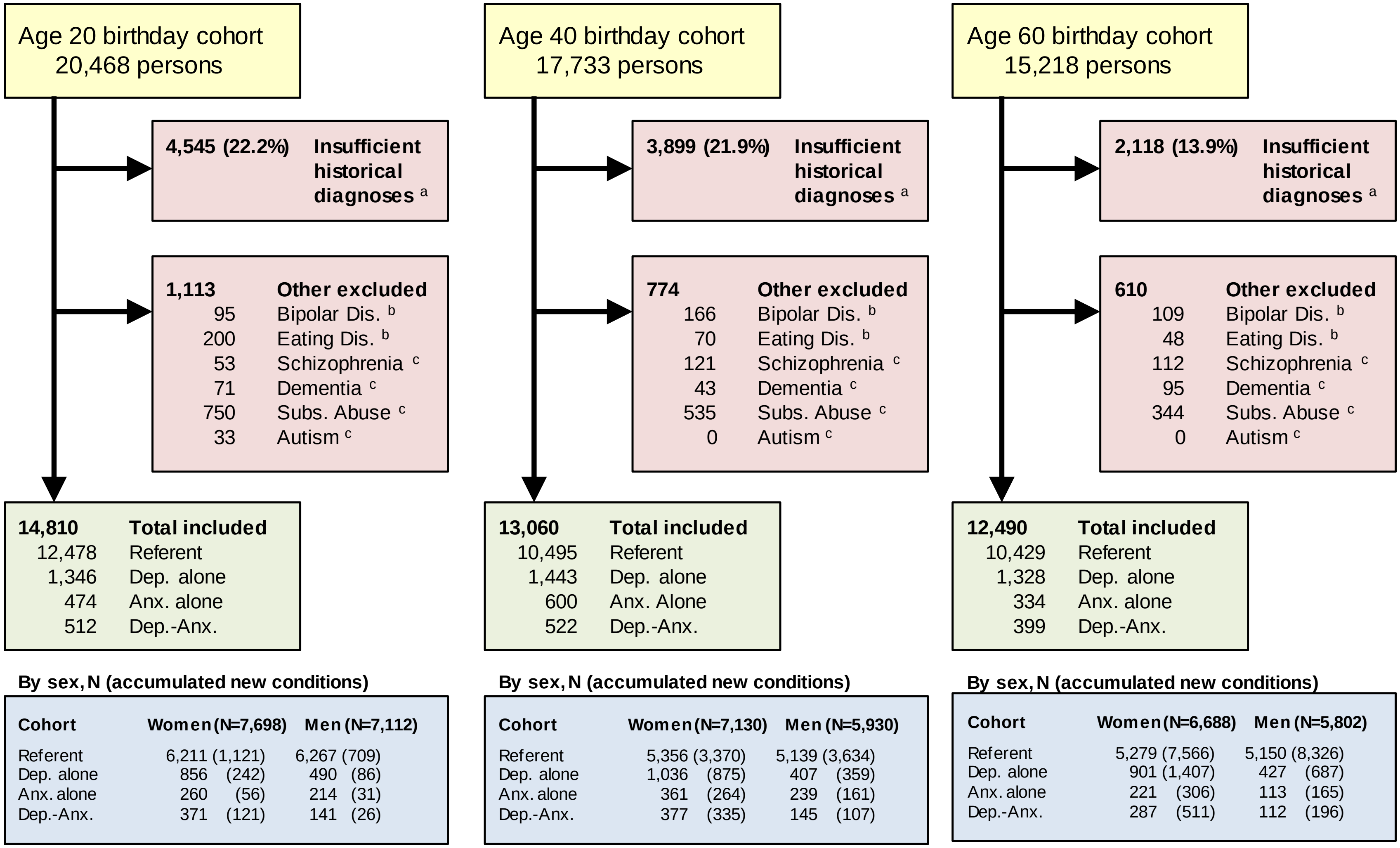

eFigure 1. The complete populations identified by the REP Census are included in the yellow boxes. Persons were subsequently excluded for either 1) insufficient data to define study variables or for 2) presence of conditions at index included in exclusion criteria (pink boxes). The numbers of persons included in final analyses within each age cohort and separately by exposure group are summarized in the green boxes. The numbers of accumulated chronic conditions that were observed during follow-up are summarized in the blue boxes separately by exposure group and separately by sex. **Abbreviations:** Anx. alone = diagnosed anxiety disorder alone (without co-occurring depressive disorder diagnoses); Bipolar Dis. = bipolar spectrum disorders; Dep. alone = diagnosed unipolar depressive disorder alone (without co-occurring anxiety disorder diagnoses); Dep.-Anx. = co-occurring diagnosed unipolar depressive and anxiety disorders; Eating Dis. = eating disorders; Subs. Abuse = substance use disorders. **Footnotes:** [a] Insufficient historical diagnoses is defined as having less than two years of diagnosis codes in the five years before the index birthdate. [b] Exclusions based on ICD-9 diagnosis code groupings. [c] Exclusions based on DHHS-defined chronic conditions code groupings.

© 2022 Bobo WV et al. *JAMA Network Open.*

eFigure 2. Balance of Characteristics by Birthday Age Cohort
2A. Balance of characteristics in the 20-year-old birthday cohort.

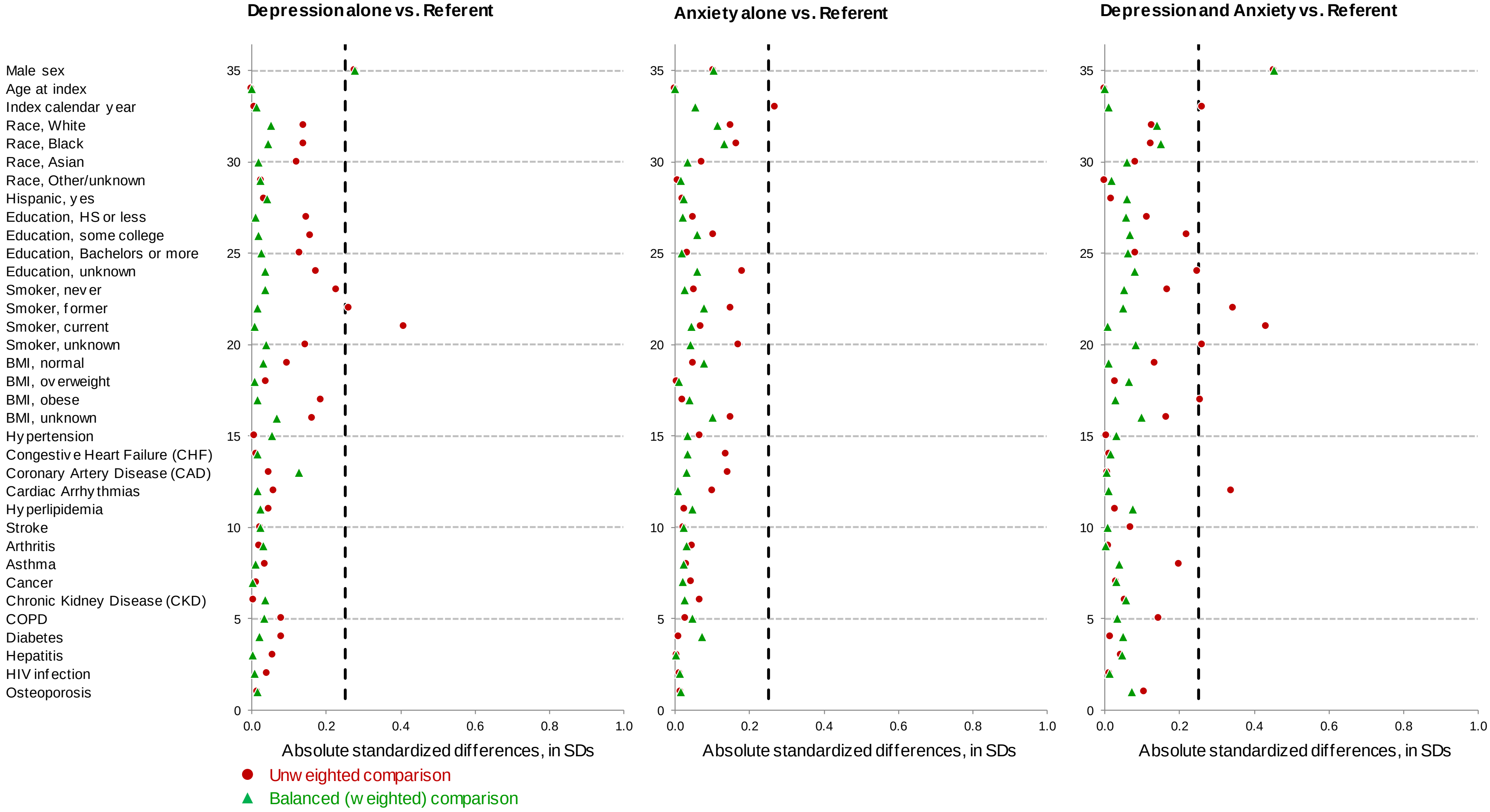

eFigure 2. To account for potential confounders associated with the accumulation of chronic conditions, adjusted hazards ratios (HRs) were calculated using inverse probability weights (IPWs) derived from generalized boosting models (GBMs). The GBMs were constructed separately for men and women and separately within each birthday age cohort (ages 20, 40, and 60 years). The covariates included in the balancing were calendar year at index date (as an integer from 2005 through 2014), race (in categories as White, Black, Asian, Other/unknown), ethnicity (Hispanic, non-Hispanic), level of education (in categories as high school or less, some college, 4 years of college or more, and unknown), smoking status (never smoker, former smoker, current smoker, and unknown), body mass index (BMI; in categories as <25, 25 to <30, ≥ 30, and unknown), and prevalence of each of the 15 chronic conditions (hypertension, congestive heart failure, coronary artery disease, cardiac arrhythmias, hyperlipidemia, stroke, arthritis, asthma, cancer, chronic kidney disease, chronic obstructive pulmonary disease, diabetes, hepatitis, HIV infection, and osteoporosis). The figure shows the pre-weighting balance of covariates at index birthday (red circles), and the post-weighting balance of covariates at index birthday (green triangles). Note that the balance of almost all covariates improves with weighting, and that none of the covariates have an imbalance of greater than 0.25 absolute standardized differences after weighting.

© 2022 Bobo WV et al. *JAMA Network Open.*

2B. Balance of characteristics in the 40-year-old birthday cohort.

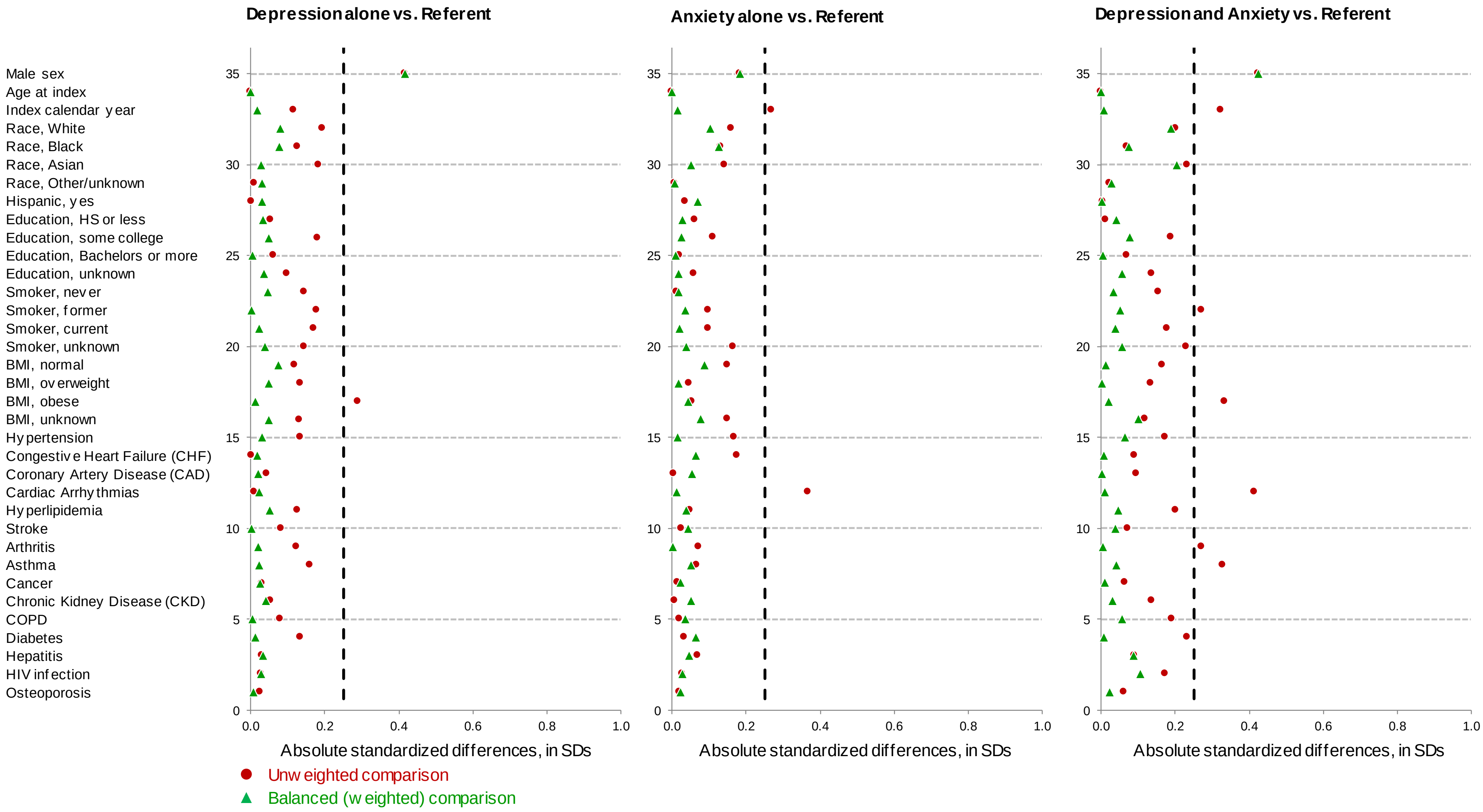

eFigure 2. To account for potential confounders associated with the accumulation of chronic conditions, adjusted hazards ratios (HRs) were calculated using inverse probability weights (IPWs) derived from generalized boosting models (GBMs). The GBMs were constructed separately for men and women and separately within each birthday age cohort (ages 20, 40, and 60 years). The covariates included in the balancing were calendar year at index date (as an integer from 2005 through 2014), race (in categories as White, Black, Asian, Other/unknown), ethnicity (Hispanic, non-Hispanic), level of education (in categories as high school or less, some college, 4 years of college or more, and unknown), smoking status (never smoker, former smoker, current smoker, and unknown), body mass index (BMI; in categories as <25, 25 to <30, ≥ 30, and unknown), and prevalence of each of the 15 chronic conditions (hypertension, congestive heart failure, coronary artery disease, cardiac arrhythmias, hyperlipidemia, stroke, arthritis, asthma, cancer, chronic kidney disease, chronic obstructive pulmonary disease, diabetes, hepatitis, HIV infection, and osteoporosis). The figure shows the pre-weighting balance of covariates at index birthday (red circles), and the post-weighting balance of covariates at index birthday (green triangles). Note that the balance of almost all covariates improves with weighting, and that none of the covariates have an imbalance of greater than 0.25 absolute standardized differences after weighting.

© 2022 Bobo WV et al. *JAMA Network Open.*

2C. Balance of characteristics in the 60-year-old birthday cohort.

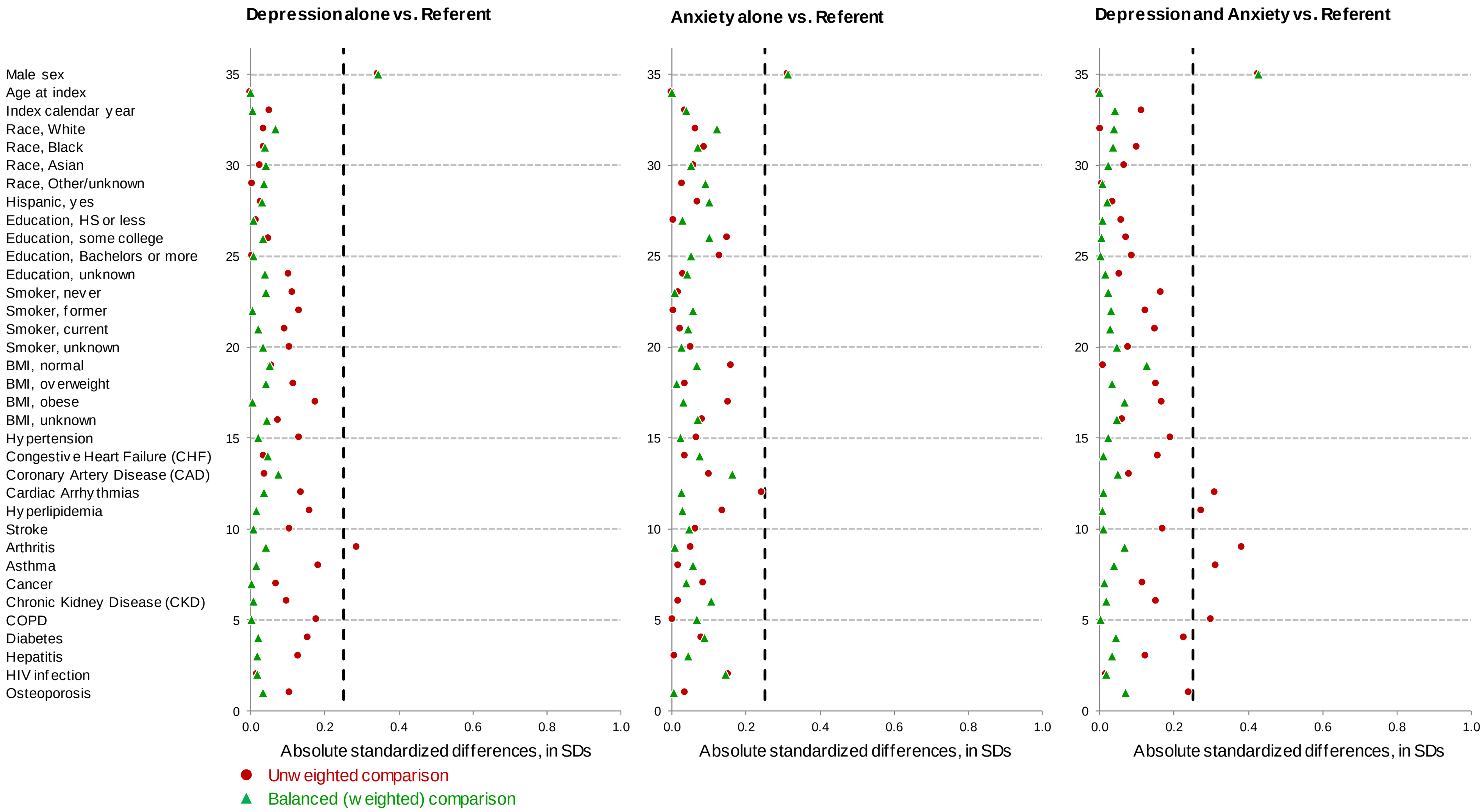

eFigure 2. To account for potential confounders associated with the accumulation of chronic conditions, adjusted hazards ratios (HRs) were calculated using inverse probability weights (IPWs) derived from generalized boosting models (GBMs). The GBMs were constructed separately for men and women and separately within each birthday age cohort (ages 20, 40, and 60 years). The covariates included in the balancing were calendar year at index date (as an integer from 2005 through 2014), race (in categories as White, Black, Asian, Other/unknown), ethnicity (Hispanic, non-Hispanic), level of education (in categories as high school or less, some college, 4 years of college or more, and unknown), smoking status (never smoker, former smoker, current smoker, and unknown), body mass index (BMI; in categories as <25, 25 to <30, ≥ 30, and unknown), and prevalence of each of the 15 chronic conditions (hypertension, congestive heart failure, coronary artery disease, cardiac arrhythmias, hyperlipidemia, stroke, arthritis, asthma, cancer, chronic kidney disease, chronic obstructive pulmonary disease, diabetes, hepatitis, HIV infection, and osteoporosis). The figure shows the pre-weighting balance of covariates at index birthday (red circles), and the post-weighting balance of covariates at index birthday (green triangles). Note that the balance of almost all covariates improves with weighting, and that none of the covariates have an imbalance of greater than 0.25 absolute standardized differences after weighting.

© 2022 Bobo WV et al. *JAMA Network Open.*

eFigure 3. Kaplan-Meier Estimates of Mortality in the Age 60 Years Cohort Separately for Women and Men

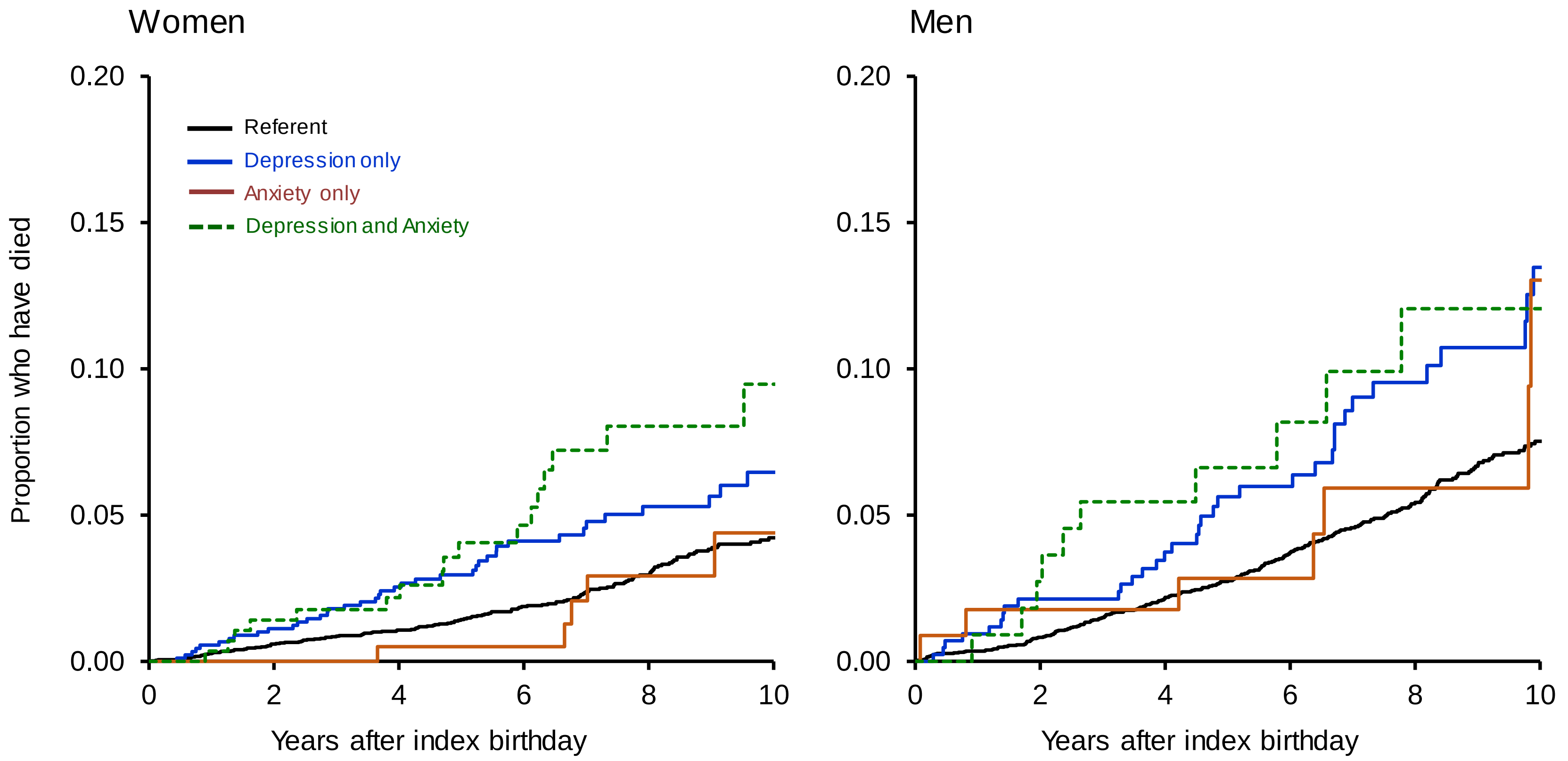

Number at risk

Referent	5,279	5,166	4,489	3,254	2,166	1,231	5,150	5,023	4,285	3,012	1,962	1,084
Depression	901	876	744	512	344	171	427	411	337	238	159	89
Anxiety	221	217	191	142	85	48	113	110	94	69	45	24
Dep-Anx	287	278	228	158	93	50	112	107	88	57	40	24

eFigure 3. The proportion of persons in the 60-year-old birthday cohort who died over 10 years of follow-up separately within the referent, depression only, anxiety only, and comorbid depression-anxiety strata. The number of persons at risk at 2-year intervals are reported below the figures. Hazards ratios from Cox proportional hazards models corresponding to these curves are reported in Supplementary Table 3.

© 2022 Bobo WV et al. *JAMA Network Open.*
